# Preparation and Preliminary Analysis of Several Nanoformulations Based on Plant Extracts and Biodegradable Polymers as a Possible Application for Chronic Venous Disease Therapy

**DOI:** 10.3390/polym16101362

**Published:** 2024-05-10

**Authors:** Andreea Roxana Ungureanu, Emma Adriana Ozon, Adina Magdalena Musuc, Mihai Anastasescu, Irina Atkinson, Raul-Augustin Mitran, Adriana Rusu, Liliana Popescu, Cerasela Elena Gîrd

**Affiliations:** 1 Faculty of Pharmacy, “Carol Davila” University of Medicine and Pharmacy, 6 Traian Vuia Street, 020956 Bucharest, Romania; andreea.ungureanu@drd.umfcd.ro (A.R.U.); liliana.popescu22@umfcd.ro (L.P.); cerasela.gird@umfcd.ro (C.E.G.); 2 Institute of Physical Chemistry—Ilie Murgulescu, Romanian Academy, 202 Splaiul Independenței, 060021 Bucharest, Romania; manastasescu@icf.ro (M.A.); iatkinson@icf.ro (I.A.); rmitran@icf.ro (R.-A.M.); arusu@icf.ro (A.R.)

**Keywords:** natural extracts, nanotechnology, poly-lactic-co-glycolic acid, polyhydroxybutyrate, X-ray diffractometry, atomic force microscopy, thermogravimetry

## Abstract

Nanotechnology is one of the newest directions for plant-based therapies. Chronic venous disease often predisposes to long-term and invasive treatment. This research focused on the inclusion of vegetal extracts from *Sophorae flos* (SE), *Calendulae flos* (CE), and *Ginkgo bilobae folium* (GE) in formulations with PHB and PLGA polymers and their physicochemical characterization as a preliminary stage for possible use in the development of a complex therapeutic product. The samples were prepared by an oil–water emulsification and solvent evaporation technique, resulting in suspensions with high spreadability and a pH of 5.5. ATR-FTIR analysis revealed bands for stretching vibrations (O-H, C=O, and C-H in symmetric and asymmetric methyl and methylene) in the same regions as the base components, but switched to high or low wavenumbers and absorbance, highlighting the formation of adducts/complexes between the extracts and polymers. The obtained formulations were in the amorphous phase, as confirmed by XRD analysis. AFM analysis emphasized the morphological peculiarities of the extract–polymer nanoformulations. It could be noticed that, in the case of SE-based formulations, the dominant characteristics for SE-PHB and SE-PLGA composition were the formation of random large (SE-PHB) and smaller uniform (SE-PLGA) particles; further on, these particles tended to aggregate in the case of SE-PHB-PLGA. For the CE- and GE-based formulations, the dominant surface morphology was their porosity, generally with small pores, but larger cavities were observed in some cases (CE- and GE-PHB). The highest roughness values at the (8 µm × 8 μm) scale were found for the following samples and succession: CE-PHB < SE-PLGA < SE-PHB-PLGA. In addition, by thermogravimetric analysis, impregnation in the matrix of compression stockings was evaluated, which varied in the following order: CE-polymer > SE-polymer > GE-polymer. In conclusion, nine vegetal extract–polymer nanoformulations were prepared and preliminarily characterized (by advanced physicochemical methods) as a starting point for further optimization, stability studies, and possible use in complex pharmaceutical products.

## 1. Introduction

The confluence between nanotechnologies and plant derivatives can be summarized in two important research directions [1]: 1. synthesis of nanomaterials using plant extracts (e.g., metallic Ag nanoparticles) [2,3]; and 2. integration into nanosystems (plant extract as a phytocomplex, or isolated active phytochemicals). Some considerations for using nanotechnologies for plant derivatives are the following: to increase bioavailability, decrease toxicity, protect against physical and chemical degradation, and ensure controlled release. Thus, various nanosystems have been developed: nanoemulsions, nanoparticles (nanospheres, nanocapsules, solid lipid nanoparticles, and nanostructured lipid carriers), liposomes (conventional, secondary, or tertiary), ethosomes, invasomes, transfersomes, niosomes, and phytosomes [4,5,6,7,8,9]. The evolvement of topical–transdermal pharmaceutical products has shown particular interest due to the expectation of better compliance (being a non-invasive treatment route). The interaction between the skin and nanoformulations has different mechanisms: adhesion to the skin surface, modification of the hydration level, structure, or/and polarity of the skin, and lipid exchange through the intercellular lipid domains. There are several ways of penetration: intercellular, transcellular, and transfollicular (nanoparticles can last up to 10 days in hair follicles [10], and even those with a size of 600 nm can have high penetrability through the hair follicles after massaging the skin [11]).

The potential of phytochemicals in the treatment of chronic venous disease (CVD) was confirmed by clinical practice guidelines [12,13,14], which highlighted flavonoids as the principal pharmacological treatment (recommendation class IIa, level A). Flavonoids proved to be effective for CVD [15], due to their multiple mechanisms to counteract vascular damage (antioxidant, anti-inflammatory, enhances capillary resistance, and inhibits matrix metalloproteinases [16,17,18]). Their bioavailability problems are known, and they are used after micronization, but a promising direction is developing: nanoformulations [19,20]. Compression treatment is a standard therapy for chronic venous disease [21]; according to guidelines, compression stockings have the recommendation class I with the level of evidence B, and the association with flavonoids has the recommendation class IIa with the level of evidence A [12,13]. 

Extracts from *Sophorae flos*, *Calendulae flos*, and *Ginkgo bilobae folium* were previously characterized (by spectrophotometric methods, FTIR-MS, and UHPLC-HRMS/MS) and proved to be rich in polyphenols (rutin, quercetin, isorhamnetin, hyperoside, ginkgolide A, B, C, sophoricoside, calenduloside E, F, G, narcissin, and apigenin) [16]. Additionally, the antioxidant activity of the extracts was previously determined by in vitro methods and correlated with their phytochemical composition [16]. The damage from chronic venous disease is multifactorial, and these phytocomplexes can prevent several pathological mechanisms simultaneously through a multimodal effect (antioxidant, anti-inflammatory, and antithrombotic) [16,22,23]. 

Polyhydroxybutyrate (PHB) is a natural biopolymer with the following main advantages: biocompatibility, high encapsulation efficiency, and the monomers resulting from its degradation (e.g., 3-hydroxybutyrate, 4-hydroxybutyrate) are easily washed-out by the organism [24]. Polylactic-co-glycolic acid (PLGA), one of the most well-known synthetic polymers, is useful for the preparation of solid polymeric nanoparticles [24]. It was used for the encapsulation of rutin (main compound in *Sophorae flos* extract [16]) to obtain nanoparticles with a size of 250–300 nm [25] and, depending on the method applied for preparation, another study led to rutin nanoparticles with sizes below 200 nm [26]. PLGA microparticles loaded with *Ginkgo bilobae* extract were developed to functionalize a hydroxyapatite matrix as a graft for bone diseases [27]. Extract from *Calendula officinalis* was incorporated into solid lipid nanoparticles which were analyzed for wound treatment. It was noted that nanoparticles with extract favored re-epithelialization (resulting in an approximately three times greater percentage of efficacy compared to the extract alone) [28].

The current study represents a preliminary stage in a broad context of the development of a product that combines pharmacological therapy (consisting of plant extracts brought into a nanoformulation with biopolymers) and compressive therapy (consisting of compression stockings). As a first approach in our project, the aim of the current research is to integrate three plant extracts (*Sophorae flos* extract—SE, *Calendulae flos* extract—CE, and *Ginkgo bilobae folium* extract—GE) in nanoformulations with PHB and PLGA polymers, and present their physicochemical analysis and an investigation of the possibility to functionalize a compression stocking matrix.

## 2. Materials and Methods

### 2.1. Preparation and pH Determination

The following were used for preparation: biopolymers PHB and PLGA (Merck Millipore, Darmstadt, Germany), lyophilized plant extracts (previously obtained [16]), chloroform and ethanol (Sigma-Aldrich, Saint Louis, MO, USA), and polyvinyl alcohol (Jakobsohn LTD, Hampshire, UK). Formulations with extracts were made by an oil–water emulsification and solvent evaporation technique [24]. The oil phase was obtained by dissolving the nanoparticle-forming polymer (PHB, PLGA, or PHB:PLGA 1:1 m/m mixture) in chloroform (0.5 g polymer in 100 mL chloroform). Chloroform is classified as a class 2 solvent [29]. The water phase was obtained by dissolving the dry extract (SE, CE, or GE) in ethanol. The extracts were analyzed in our previous work and are characterized by their main components, quantified by UHPLC-HRMS-MS: 104,186.77 μg rutin/g SE, 97,049.32 μg isorhamnetin/g SE, 46,678.34 μg quercetin/g SE, 5032.60 μg isorhamnetin/g GE, 4504.66 μg quercetin/g GE, 3907.47 μg rutin/g GE, 20,676.63 μg chlorogenic acid/g CE, 11,286.93 μg isorhamnetin/g CE, and 2165.42 μg rutin/g CE [16]. A polymer:extract ratio of 1:1 (m/m) was used. The two phases were mixed under magnetic stirring at 700 rpm for 5 min. Subsequently, the sample was added to 100 mL of 3% polyvinyl alcohol and shaken with a mechanical shaker for 2 min in an ice bath. The mixture was then brought under a magnetic stirrer (Heidolph MR 3001 K) at 1000 rpm for 2 h. The samples were conditioned at 2–8 °C. The pH determination was performed using indicator paper (Merck Millipore, Darmstadt, Germany) and pH scale as indicated by the manufacturer.

### 2.2. Spreadability Measurement

The Ojeda–Arbussa extensiometric method was applied using an extensiometric device consisting of two overlapping square plates, with millimeter graduation on the external side of the lower plate. A 0.5 g sample was placed on the lower plate, over which the upper plate was added. The diameter of the spread sample was measured after being pressed under the weight of the plate (150 g). At intervals of one minute, additional weights (100 g, 200 g, 300 g, 400 g, 500 g, and 600 g) were placed on the upper plate, and the diameters of the formed circles were determined [30]. The surface on which the sample was extended under the action of the weights was calculated.

### 2.3. Attenuated Total Reflectance Fourier Transform Infrared Spectroscopy (ATR-FTIR) Analysis

The ATR-FTIR analysis was performed using a JASCO FTIR-4200 spectrophotometer with an ATR-PRO450-S accessory (Tokyo, Japan), recording spectra in the range of wavenumbers 4000–400 cm^−1^ at a 4 cm^−1^ resolution. Spectra are presented as variations of absorbance as a function of wavenumbers. This method is known for having the following advantages: liquid samples can be analyzed, a small amount of sample is necessary, reduced analysis time, good repeatability, and high sensitivity. The application of ATR does not require any previous processing of the sample and can be successfully applied for oils or plant extracts. The spectra are generally divided into four regions: 4000–2500 cm^−1^ (single bonds domain), 2500–2000 cm^−1^ (triple bonds domain), 2000–1500 cm^−1^ (double bonds domain), and <1500 cm^−1^ (fingerprint) [31,32,33].

### 2.4. X-ray Diffractometry (XRD)

The XRD analysis was performed by a Rigaku Ultima IV diffractometer (Rigaku Co., Tokyo, Japan), operated in parallel beam geometry, using CuKα radiation (λ = 1.5406 Å). Other parameters for analysis were the divergent slit size, 1.00 mm; divergent height-limiting slit, 10 mm; 2θ range, between 10° and 60°; and speed of 2°/min at a step size of 0.02° [34]. The assay was performed on formulations as solid polymeric films obtained after drying a thin layer of formulation at room temperature (20 ± 5 °C) for 24 h. The results are presented as diffractograms.

### 2.5. Atomic Force Microscopy (AFM)

The AFM measurements were performed by an XE-100 atomic force microscope (Park Systems Corporate, Suwon, Republic of Korea) with decoupled XY/Z scanners. The main component was NSC36B tips (MikroMasch, Sofia, Bulgaria) operating in non-contact mode and characterized by the following description: a typical radius curvature less than 8 nm, 40° full cone angle, 1 μm thickness, 15 μm height, 90 μm length, 32 μm width, and 130 kHz resonance frequency. The images were processed using the XEI program (v 1.8.0, Park Systems Corporate, Suwon, Republic of Korea) and presented in “enhanced contrast” mode. The AFM images are accompanied by representative surface profiles, i.e., line-scans collected along the horizontal direction, emphasizing surface characteristics at the nm scale [35,36,37,38,39].

### 2.6. Thermogravimetry

Thermogravimetric analyses were conducted using a Mettler Toledo TGA/SDTA851e thermogravimeter (Mettler-Toledo, Greifensee, Switzerland) under the following parameters: synthetic air flow under 80 mL min^−1^, and a heating rate of 10 °C min^−1^ [39]. This method was applied to samples consisting of compression stockings (pieces of 2 × 2 cm^2^), used as a matrix, each immersed in a nanoparticle suspension for 30 min.

## 3. Results

### 3.1. Preparation and pH Determination

**Figure 1:**
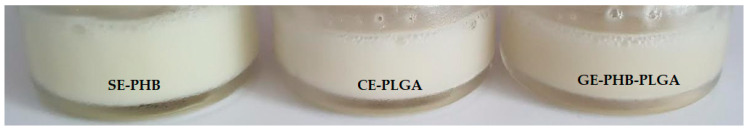
Formulations with extracts and biodegradable polymers: SE = *Sophorae flos* extract, CE = *Calendulae flos* extract, GE = *Ginkgo bilobae folium* extract, PHB = polyhydroxybutyrate, PLGA = polylactic-co-glycolic acid.

The obtained formulations consisted of particles suspended in polyvinyl alcohol solution with a homogeneous appearance (Figure 1). Phase separation phenomena occurred, but redispersed quickly after vigorous stirring (30 s). All samples had a pH of 5.5, corresponding to the physiological pH of skin.

### 3.2. Spreadability Assay

The obtained formulations have high spreadability. The results are shown in Table 1 and their variations, depending on the type of extract and polymer, are presented in Supplementary Materials. 

Related to the final surface on which the sample was extended, the highest spreadability belonged to the sample with *Caledulae flos* extract and PHB (141.72 ± 7.24 cm^2^), and the lowest belonged to the sample with *Sophorae flos* extract and PLGA (73.36 ± 0.87 cm^2^). Related to the type of polymer, both for the samples with PHB and for those with PLGA, and also for the mixture of the two polymers, the spreading capacity varied in the order of CE > GE > SE. Regarding the extracts, for each individual extract the variation followed the sequence PHB > PHB-PLGA > PLGA. 

The results suggest a rapid spread of the samples on both the skin and the textile material of the compression stocking, as indicated by their fluid nature. From this analysis, it can be concluded that the formulations with PHB have the highest spreadability, the association with PLGA leading to its decrease (the PHB-PLGA formulations values being intermediate between PHB and PLGA). Among the extracts, CE leads to formulations with the best spreadability. 

### 3.3. Attenuated Total Reflectance Fourier Transform Infrared Spectroscopy (ATR-FTIR) Analysis

Both the spectra of the formulations and those of the base components (extracts and biopolymers) were successfully recorded (Supplementary Materials). ATR-FTIR spectroscopy was employed to analyze the chemical composition and functional groups present in the formulations. By comparing the infrared spectra of the formulations with those of pure PHB and PLGA, we aimed to identify any changes or interactions occurring due to the incorporation of plant extracts. This characterization helped elucidate the molecular interactions between the polymers and the active components of the plant extracts, providing valuable information on formulation stability and compatibility.

Vegetal extracts’ spectra revealed a wide band in the 4000–3000 cm^−1^ region, characteristic for stretching vibrations of O-H bonds from alcohols and phenols (SE peak at 3407.9 cm^−1^, CE peak at 3401.6 cm^−1^_,_ and GE peak at 3398.4 cm^−1^). In addition, biopolymers exhibited bands in this region, with a PHB peak at 3430.1 cm^−1^ and PLGA peak at 3645.4 cm^−1^_,_ with an additional peak for PLGA (3500.1 cm^−1^) in the 3550–3450 cm^−1^ region, corresponding to stretching vibration of the dimeric OH bond. Asymmetric stretching vibrations of C-H bonds from methyl groups (2970–2950 cm^−1^ [31]) were observed for PHB (2963.1 cm^−1^) and GE (2961.5 cm^−1^), and vibrations from methylene groups (2935–2915 cm^−1^ [31]) were found for GE (2926.6 cm^−1^) and SE and CE (2923.5 cm^−1^). Symmetric stretching vibrations of C-H bonds from methyl groups (2880–2860 cm^−1^ [31]) were revealed for biopolymers (PHB peak at 2866.5 cm^−1^ and PLGA peak at 2879.2 cm^−1^) and from methylene groups (2865–2845 cm^−1^ [31]) for PLGA (2857 cm^−1^) and GE and CE (2850.7 cm^−1^). 

In the double bond region (2000–1500 cm^−1^), peaks characteristic for stretching vibrations of C=O bonds (1780–1650 cm^−1^ [31]) were found for PLGA (1761.5 cm^−1^), PHB (1733 cm^−1^), and GE (1698.2 cm^−1^); of C=C bonds from alkenyl groups (1680–1620 cm^−1^) or conjugated quinones or ketones (1650–1600 cm^−1^ [31]) for SE (1650.7 cm^−1^and 1612.7 cm^−1^) and CE and PLGA (1625.3 cm^−1^). Other peaks from natural extracts corresponded to C=C conjugated bonds (around 1600 cm^−1^ [31]) for GE (1609.5 cm^−1^) and to aromatic rings (around 1500 cm^−1^ [31]) for SE (1514.5 cm^−1^) and GE (1508.2 cm^−1^). 

**Figure 2:**
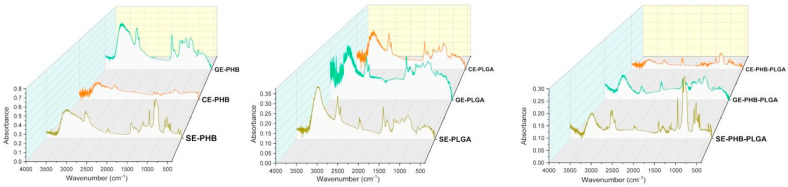
ATR-FTIR spectra of formulations: SE = *Sophorae flos* extract, CE = *Calendulae flos* extract, GE = *Ginkgo bilobae folium* extract, PHB = polyhydroxybutyrate, PLGA = polylactic-co-glycolic acid.

The ATR-FTIR spectra of the formulations (Figure 2) revealed bands at the same regions as the base components, but with a few differences in wavenumber and absorbance, influenced by the interactions between components. 

Stretching vibrations of O-H bonds from alcohols and phenols characterized the following formulations: GE-PHB (3401.6 cm^−1^), CE-PHB (3436.4 cm^−1^), SE-PLGA (3433.2 cm^−1^), SE-PHB-PLGA (3449.1 cm^−1^, 3072.3 cm^−1^, 3043.8 cm^−1^), and CE-PHB-PLGA (3433.2 cm^−1^); vibrations of dimeric O-H bonds characterized SE-PHB (3461.7 cm^−1^), GE-PLGA (3488.8 cm^−1^), CE-PLGA (3455.4 cm^−1^), and GE-PHB-PLGA (3468.1 cm^−1^); and vibrations of O-H bonds from acids characterized SE-PHB (2663.3 cm^−1^) and CE-PHB and GE-PHB-PLGA (2660.2 cm^−1^). Regarding C-H bonds, formulation peaks from stretching vibrations were classified as follows: 1. asymmetric from methyl groups: SE-PHB (2958.3 cm^−1^), CE-PLGA and SE-PHB-PLGA (2955.1 cm^−1^), and CE-PHB-PLGA (2967.8 cm^−1^); 2. asymmetric from methylene groups: SE-PHB, CE-PHB-PLGA (2920.3 cm^−1^), GE-PHB (2933 cm^−1^), CE-PHB and CE-PLGA (2926.6 cm^−1^), and SE-PLGA, GE-PLGA, and GE-PHB-PLGA (2929.8 cm^−1^); 3. symmetric from methyl groups: SE-PLGA (2860.2 cm^−1^) and 4. symmetric from methylene groups: SE-PLGA and GE-PHB (2860.2 cm^−1^), GE-PLGA and SE-PHB-PLGA (2853.8 cm^−1^), CE-PLGA (2850.7 cm^−1^), and CE-PHB-PLGA (2863.3 cm^−1^).

In the double bonds region, stretching vibrations of C=O were observed for SE-PHB (1736.1 cm^−1^), SE-PLGA and CE-PLGA (1745.6 cm^−1^), and GE-PHB, CE-PHB, GE-PLGA, SE-PHB-PLGA, and GE-PHB-PLGA (1739.3 cm^−1^); vibrations of C=C bonds or conjugated quinones/ketones were found for all formulations (values around 1650 cm^−1^) with the exception of CE-PHB-PLGA, and only for SE-PHB (1603.2 cm^−1^) were there noticed vibrations of C=C conjugated bonds.

Each extract brings changes in the position and intensity of the polymer bands depending on the phytocompounds contained and the way they interact with the polymers; thus, each formulation has an individualized spectrum. Among all polymeric formulations, the greatest reduction in intensity was noticed for *Calendulae flos* extract, possibly because of the high content of chlorogenic acid [16], which may be involved in more interactions with the polymers by -COOH group and decrease the intensity of -OH peaks.

### 3.4. X-ray Diffractometry (XRD)

**Figure 3:**
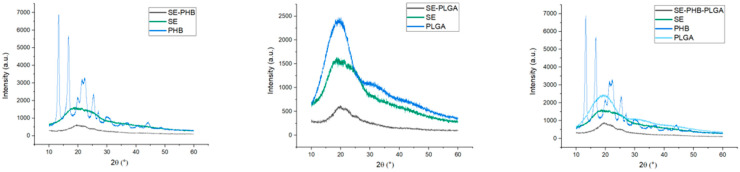
XRD analysis results for SE formulations and their base components.

Diffractograms for SE formulations in comparison with base components’ diffraction patterns are presented in Figure 3, and those belonging to the other two extracts (GE and CE) are shown in Supplementary Materials. X-ray diffraction analysis was utilized to investigate the crystalline structure of the formulations. PHB and PLGA polymers exhibit varying degrees of crystallinity, which can influence their mechanical properties and stability. By analyzing the XRD patterns, we aimed to assess any changes in crystallinity induced by the incorporation of plant extracts and the preparation method employed. This characterization provided insights into the physical state and homogeneity of the formulations, aiding in optimizing their performance and efficacy.

Among all the samples analyzed, the only diffractogram showing defined peaks corresponds to PHB, which is considered to be in a crystalline state (with a crystallinity degree of 82.5%). The other components of the formulations are in an amorphous or partially crystalline state. Regarding the formulations, for SE-PHB the crystallinity of PHB faded, resulting in a pattern without peaks and with a large hump at reduced intensity (640 u.a., around 20°), confirming the amorphous phase after extract–polymer interaction. Analogously, large humps around 20° with reduced intensity were noted for SE-PLGA (630 u.a.) and SE-PHB-PLGA (906 u.a.), suggesting an amorphous phase. However, a higher intensity was noted for the SE-PHB-PLGA formulation compared to SE-PHB and SE-PLGA.

### 3.5. Atomic Force Microscopy (AFM)

**Figure 4:**
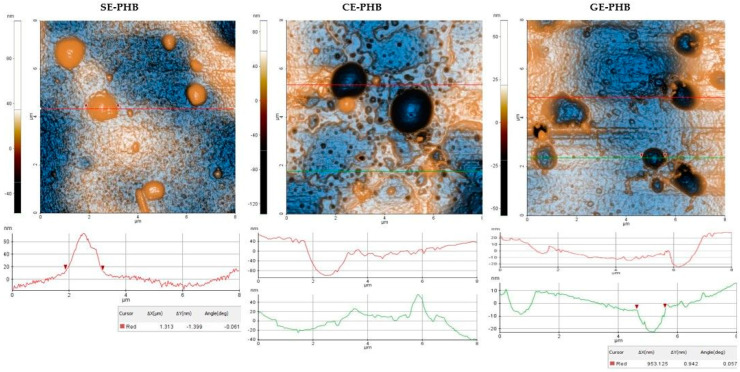
2D AFM images of PHB formulations: SE = *Sophorae flos* extract, CE = *Calendulae flos* extract, GE = *Ginkgo bilobae folium* extract, PHB = polyhydroxybutyrate, red and green lines = surface profiles.

**Figure 5:**
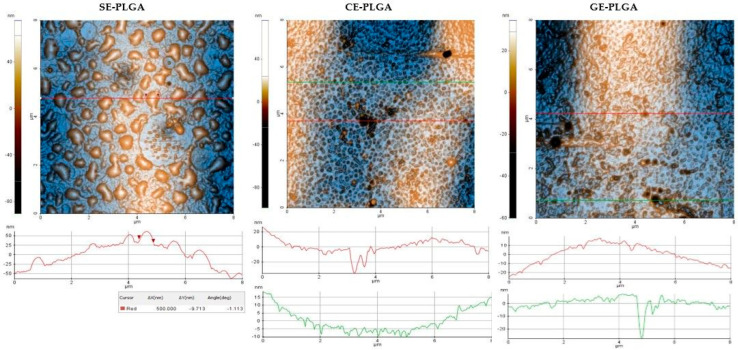
2D AFM images of PLGA formulations (SE = *Sophorae flos* extract, CE = *Calendulae flos* extract, GE = *Ginkgo bilobae folium* extract, PLGA = polylactic-co-glycolic acid, red and green lines = surface profiles).

**Figure 6:**
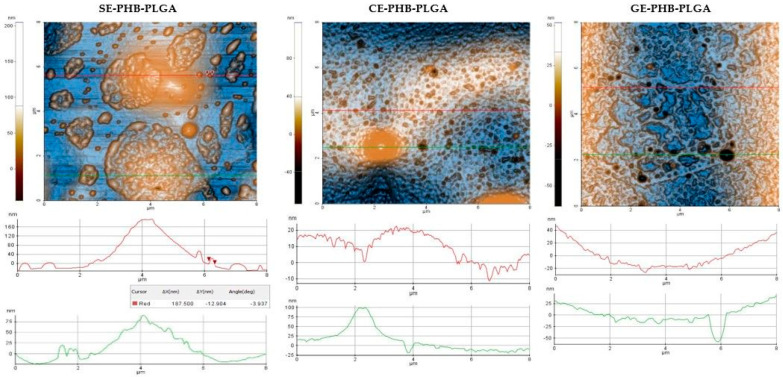
2D AFM images of PHB-PLGA formulations (SE = *Sophorae flos* extract, CE = *Calendulae flos* extract, GE = *Ginkgo bilobae folium* extract, PHB = polyhydroxybutyrate, PLGA = polylactic-co-glycolic acid, red and green lines = surface profiles).

**Figure 7:**
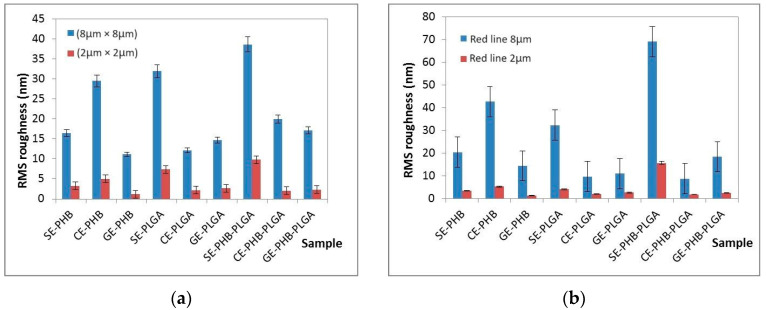
RMS roughness of all formulations estimated from whole images (**a**) and from the red line (**b**).

Results are presented as 2D enhanced-contrast AFM topographic images, scanned at the scale of (8 × 8) μm^2^, accompanied by representative line profiles, in Figure 4, Figure 5, Figure 6 and Figure 7. Other recorded AFM images (3D (8 × 8) μm^2^ and (2 × 2) μm^2^) are shown in Supplementary Materials.

Some differences can be noticed between the AFM appearance of PHB formulations (Figure 4): SE-PHB appears more compact and uniform with narrow surface nano-pores, as can be observed from the “jagged” aspect of the surface profile plotted below the SE-PHB AFM image. CE-PHB and GE-PHB show slightly larger pores (tens of nm in diameter), these being more uniformly distributed in CE-PHB samples, together with cavities (>900 nm in diameter and up to 100 nm in depth). Randomly, large particles are seen on SE-PHB and, less protrudingly, on CE-PHB, while in the case of GE-PHB they are less visible (most of them being nanoparticles).

Roughness parameters for PHB formulations (Rpv—peak to valley, Rq—RMS roughness, and Ra—average roughness) (see Table 2 and Figure 7a) decreased in the following order: CE-PHB > SE-PHB > GE-PHB. The same succession was observed at (8 μm × 8 μm) and (2 μm × 2 μm), and even for the roughness evaluated along the red line (Figure 7b). RMS roughness (Rq) values of CE-PHB were approximately 2–3 times higher than SE-PHB (1.79) and GE-PHB (2.63), indicating that CE-PHB had a more corrugated surface.

The AFM images of the SE-PLGA formulations (Figure 5) depict large nanostructures with uneven dimensions (different diameters on the two axes) of 400–500 nm, as well as small nanoparticles (around 10 nm). The dominant characteristic of the CE- and GE-PLGA is the surface porosity (nm-scale pores; for example, 30–40 nm) coexisting with small nanoparticles (around 20 nm). At the same points, the pores tended to unite, forming ditches/grooves (tens of nm in depth). 

Among the PLGA formulations, roughness parameters (see Table 3 and Figure 7a) showed high values for SE-PLGA compared with GE- and CE-PLGA, especially for root mean square (Rq) (2.16/2.65 times larger), indicating enhanced texture variability. In this series, the sequence of RMS roughness was SE-PLGA > GE-PLGA > CE-PLGA. In the case of CE-PLGA, at the 8 × 8 μm^2^ scale, the Rpv parameter exhibited the highest value, but not for the 2 × 2 μm^2^ scale, confirming the scale-dependence of roughness parameters and the fact that the formulations could be more uniform in smaller, localized regions outside of large textural features.

For the SE-PHB-PLGA formulations, AFM analysis (Figure 6) revealed a mixture of large parcels composed of aggregated particles and individual nanoparticles (around 200 nm). CE-PHB-PLGA showed a nano-scale porous surface with small cavities and predominantly small nanoparticles (around 20 nm) and rarely large nanoparticles (around 100 nm). GE-PHB-PLGA exhibited large surface cavities and small nanoparticles (around 20 nm).

Regarding the PHB-PLGA formulations (Table 4), the highest values of roughness parameters (Rpv, Rq, and Ra) were assigned to SE-PHB-PLGA, suggesting significant height variation across the surface. CE-PHB-PLGA and GE-PHB-PLGA showed similar values for Rq and Ra roughness parameters that were ~2 times lower than for the SE-PHB-PLGA formulations.

### 3.6. Thermogravimetric Analysis

**Figure 8:**
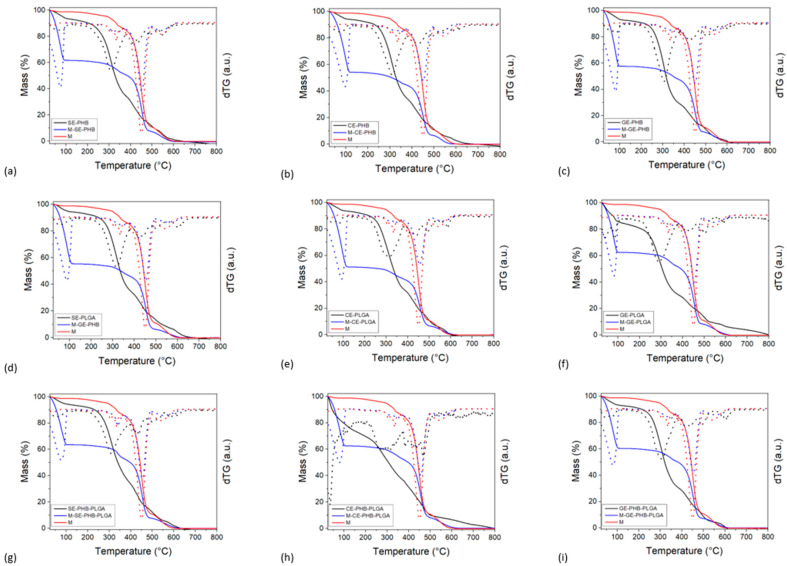
Thermogravimetric analyses results: full lines = TGA data, dotted lines = dTG data (derivative thermogravimetry), SE = *Sophorae flos* extract, CE = *Calendulae flos* extract, GE = *Ginkgo bilobae folium* extract, PHB = polyhydroxybutyrate, PLGA = polylactic-co-glycolic acid, M = compression stockings matrix, (**a**–**c**) PHB formulations, (**d**–**f**) PLGA formulations, and (**g**–**i**) PHB-PLGA formulations.

The results from thermogravimetric analyses are presented in Figure 8. Thermogravimetric analysis (TGA) was conducted to evaluate the thermal stability and decomposition behavior of the formulations. It is well known that PHB and PLGA polymers undergo thermal degradation at elevated temperatures, which can impact their processing, storage, and shelf-life. By subjecting the formulations to controlled heating, we aimed to determine their thermal stability and degradation behavior. This characterization facilitated the identification of suitable processing conditions and storage parameters to maintain formulation integrity and functionality. The matrixes (compression stocking pieces of 2 × 2 cm^2^) exhibited three different mass-loss events, between 280–380 °C, 380–500 °C, and 500–620 °C. The highest mass loss was noticed during the second combustion event. The extract–polymer formulations showed a gradual mass loss between 25 and 115 °C, which can be ascribed to the loss of volatile compounds (water, solvents, etc.). The combustion of the organics occurred from 200–250 °C to 650 °C for all samples. This combustion is made up of several superimposed events.

The matrix–formulation samples’ behavior resembled the thermal behavior of the matrix material, indicating that the matrix is the major component by weight. The solvent and volatiles’ mass-loss event was higher for the matrix–formulation samples with respect to both the matrix and natural extract–polymer samples, varying between 35% and 50%. The natural extract–polymer content was computed from the mass-loss values at 380 °C, concerning the dry sample mass at 115 °C (Table 5).

All samples exhibited between 4.9 and 9.5 %wt. formulation, with the lowest value obtained for GE-PHB-PLGA and the highest for CE-PHB. The formulation samples containing PHB had the highest overall matrix (compression stocking material) impregnation capacities (between 7.0 and 9.5 %wt).

## 4. Discussion

The current work complements the previous studies, through which three plant extracts (SE—*Sophorae flos* extract, CE—*Calendulae flos* extract, and GE—*Ginkgo bilobae folium* extract) were obtained and characterized in the context of being valuable remedies for vascular damage [16]. All three extracts were integrated into nanoformulations using biodegradable polymers (PHB and PLGA) by an oil–water emulsification and solvent evaporation technique. The obtained formulations were preliminarily analyzed by an extensiometric method (for spreadability measurements) and by advanced physicochemical methods: ATR-FTIR and XRD, in comparison with their base components (extracts and polymers); AFM for morphology; and TGA for interaction with compressive stocking material as a possible impregnation matrix.

Starting from natural extracts (SE, CE, and GE) and polymers (PHB and PLGA), nine nanoformulations were obtained. These extracts proved to be rich in flavonoids, with a remarkable percentage of flavonoids content for *Sophorae flos* extract (37.45%) [16]. Previous analysis of the extracts by quantitative UHPLC-HRMS-MS revealed that the most-abundant flavonoids for SE are rutin > isorhamnetin > quercetin > kaempferol > hesperetin; those for GE are isorhamnetin > quercetin > rutin > hyperoside > kaempferol; and those for CE are isorhamnetin > rutin > hyperoside > quercetin [16]. Most of the reported studies in the literature aimed to integrate isolated polyphenolic compounds in various nanoformulations. In the current study, whole extracts were included in formulations with PHB and PLGA and also with a polymer mixture (PHB:PLGA 1:1). The interactions with polymers can be more complex for whole extracts (defined as a mixture of phytoconstituents) in comparison with isolated compounds.

Spreadability measurements, regarding polymers, revealed the best results for PHB formulations. Even if PHB does not exhibit excellent elastic properties, association with polyvinyl alcohol (PVA), used for these formulations, probably enhances its extensibility. PVA can act as a plasticizer via hydrogen bonds which can disrupt the intermolecular forces within the polymer matrix [40,41]. Intermediate spreadability results were registered for PHB-PLGA mixture (1:1) formulations. The presence of PLGA could potentially hinder the extensibility of PHB by impairing its ability to deform and elongate. Reactive groups, such as hydroxyl (-OH) and carboxyl (-COOH), of the extracts’ phytochemicals can interact with polymeric chains, influencing spreadability. Steric interactions are also a possible mechanism through which spatial arrangements of phytocompounds’ molecules can affect the conformation and flexibility of polymer chains [42,43]. Thus, the spreadability of the formulations is influenced by the extracts’ composition, and the best results were recorded for *Calendulae flos* extract formulations.

By comparatively (base compounds vs. nanoformulation) evaluating the ATR-FTIR-obtained spectra, changes in the intensity of the peaks (3D graphs in Supplementary Materials) can be observed. Considering the extract as a phytocomplex, interactions between extracts and biopolymers are variable (depending on individual composition), leading to changes in vibrational levels. At the same time, shifts of the peaks at higher and lower wavenumbers are noted. For example, in the region 4000–2500 cm^−1^, the characteristic peaks of O-H vibration for SE (3407.9 cm^−1^) and for PHB (3430.1 cm^−1^) both have a lower value compared to the SE-PHB nanoformulation peak (3461.7 cm^−1^), suggesting the possibility of a stronger bond that requires more vibrational energy (a more stable structure); presumably, the interaction reduces the number of hydrogen bonds compared to PHB. Additionally, this can be explained based on the large molecules of natural compounds which can produce a steric effect and prevent bond vibrations. As described for SE-PHB, this also applies to CE-PHB, but for GE-PHB the observations are different. The resulting peak for the GE-PHB nanoformulations has a shift to lower wavenumbers (3401.6 cm^−1^) compared to the PHB peak (3430.1 cm^−1^), but is higher compared to the GE peak (3308.4 cm^−1^). By mixing the two components (GE and PHB), more hydrogen bonds can be exposed compared to the initial ones of GE, but less compared to PHB. The same observations as in the GE-PHB case can be associated with all nanoformulations of PLGA. All these peak shift phenomena suggest the formation of complexes or adducts between the extracts and biopolymers.

There are studies which have analyzed the physicochemical behavior of quercetin (a flavonoid found in all three extracts) in formulations with polymers. Inclusion in PLGA nanosystems did not change the characteristics of the polymers [44]. A study that followed the production of polymeric (PLGA) nanoparticles with quercetin and catechin found that the presence of the flavonoid compounds specifically modifies the electrical charge of the polymeric particles, but does not affect the stability in aqueous dispersions [45]. DRIFT (Diffuse Reflectance of Infrared by Fourier Transform) analysis revealed that both flavonoids interact with PLGA through hydrogen bonds, but quercetin also associates with the PLGA polymer through interactions between the carbonyl and carboxyl groups [44,45], and analysis confirmed the encapsulation of the two compounds inside the nanoparticles [45]. By FTIR analysis, polyphenols usually have a stretching band corresponding to the hydroxyl group (at 3400 cm^−1^). The analysis of PHB–quercetin microfibers suggested interactions as hydrogen bonds between the antioxidant groups and the carbonyl groups of PHB that can prevent the stretching vibrations of the hydroxyl group [46]. Other studies concentrated on the integration of components from *Ginkgo biloba* extract (bilobalide, ginkgolides A, B, and C) into mPEG-PLGA-mPEG (PELGE)-based, extended-release nanosystems for parenteral administration [47]. The absence of ginkgolides’ peaks and those corresponding to PELGE, and a shift of the peak for Pluronic F68 (used as a surfactant), observed on the thermograms of nanoparticles with phytoconstituents suggested dispersive interactions between phytocompounds and polymers [47]. Together with flavonoids, chlorogenic acid is an important compound found in *Calendulae flos* extract (quantified by UHPLC-HRMS-MS analysis with a value of 20,676.63 μg/g of extract) [16]. Polymeric (PLGA/PVA) nanoparticles with chlorogenic acid were developed for the treatment of lung infections. During characterization, it was observed that the chlorogenic acid molecules were preferentially arranged in the PLGA matrix compared to the aqueous PVA solution. Moreover, a significant release of chlorogenic acid from the nanosystem was observed and justified by the superior wetting capacity of PVA, which favors its solubility. The initial phase can be attributed to the chlorogenic acid molecules at the outer surface of the polymer nanoparticles, but after that, PLGA favors a prolonged release by matrix erosion (up to four hours). There were no interactions observed between individual polymers and chlorogenic acid (FTIR bands located at individual position) [48].

Regarding the triple bonds region (2500–2000 cm^−1^), a double band was observed in the 2300–2400 cm^−1^ area for all analyzed samples. The presence of these bands can be attributed to traces of CO_2_ in the samples as a result of CO_2_ presence in the atmosphere during the analysis [49]. In this case, it does not represent a significant interference because the region generally characterizes cyan, cyanate, isocyanate, thiocyanate, and nitrile-type functional groups [31]. These groups are neither specific to the extracts (polyphenolic compounds were identified through the UHPLC-HRMS and FTIR screenings carried out in our previous work [16]), nor specific to the used biopolymers (PHB and PLGA have well-defined formulas, without S and N atoms in their molecules). The <1500 cm^−1^ region, representing the fingerprint area, has a characteristic appearance for each extract, polymer, and nanoformulation. A multitude of peaks and bands of C-H bond vibration was observed, as well as C-O-C and C-O groups of flavonoids (1164–1042 cm^−1^, as reported in other studies [50]). 

XRD results from the current study noted the following peaks for PHB: 13.3° (6887 a.u.), 16.72° (5699 a.u.), 21.34° (3236 a.u.), 25.38° (2404 a.u.), 29.92° (1120 a.u.), and 44.22° (806 a.u.). These results are consistent with other reports in the literature which established that PHB may have an orthorhombic crystal structure (with six peaks: 13.6°, 17.3°, 22.5°, 26.8°, 29.8°, and 45.1°) [51]. The PLGA diffractogram revealed a broad hump in the region 10–30°, suggesting a partially crystalline phase (possibly masked peaks in the 20° region), but the literature mentions that even if glycolic acid imprints crystallinity, its copolymers are amorphous [52,53]; thus, PLGA is considered amorphous. Plant extracts, being mixtures of macromolecules, can have an amorphous or partially crystalline structure, depending on the structures of their phytocompounds. In this study, the diffractograms for SE, GE, and CE did not show well-defined peaks but only a broad hump between 10° and 30°, like PLGA, but at lower intensities; thus, the extracts were considered amorphous. From extract-polymer interactions resulted amorphous formulations. The lack of crystalline molecular organization can be an advantage through better solubilization and appropriate release of the active component compared to crystalline formulations [54]. In other studies, through DSC and XRD analysis, it was observed that both quercetin and catechin interact weakly with the PLGA polymer matrix, being dispersed in the polymer matrix under a non-crystalline phase [45].

AFM analysis was performed on all nine formulations, describing their morphology and roughness parameters. A rougher surface can, on the one hand, increase adherence properties and, on the other hand, establish more active sites by its increased surface area. However, in some cases, it can be a disadvantage because of decreased mechanical stability. For example, at the scale of (8 µm × 8 μm), the SE-PHB-PLGA nanoformulation was characterized by aggregative phenomena, leading to the following RMS roughness sequence: CE-PHB < SE-PLGA < SE-PHB-PLGA. Instead, along the selected surface profiles (red-lines of 8 μm, plotted near the corresponding AFM images), the order is changed between the SE and CE formulations as follows: SE-PLGA < CE-PHB < SE-PHB-PLGA

Previous studies based on the development of textile fibers with antimicrobial effects analyzed nanoemulsions with extracts of *Moringa oleifera* and *Aegle marmelos* that were impregnated on textile fibers [55]. Due to their hydrophobic phase, nanoemulsions formed an oily monolayer on the surface of the textile; the impregnation mechanism is based on the zeta potential’s neutralization at the surface, resulting in a slight reduction in tensile strength [55]. At the same time, the cotton fibers of the textile are sensitive to acids, and the slightly acidic pH (5.0–6.0) of the nanoemulsions contributed to the reduction of tensile strength. Even though the textile finishing process itself reduces tensile strength, the resistance was satisfactory [55]. In the current study, the obtained nanoformulations had a pH of 5.5. It is possible that the previously mentioned pH-dependent mechanism could be involved in the penetration of the nanoparticles inside the material of the compression stockings. In addition, other studies investigated the impregnation of textile material with tetrahydroxy–curcumin-derivative nanoemulsions for wound care [56,57]. In the thermogravimetric analysis performed in this research, the best impregnation capacity was noted for PHB nanoformulations (CE-PHB > SE-PHB > GE-PHB) and, regarding the extracts, the lowest was observed for GE nanoformulations and the best for CE (CE-PHB > CE-PLGA > CE-PHB-PLGA). A better adhesion of the CE-PHB and SE-PHB-PLGA nanoformulations to the compressive stocking matrix can be attributed to the higher roughness observed by AFM analysis for these formulations compared to the others. Further studies could consider the interactions between the obtained nanoformulations and the fibers of the compression stocking material.

From reported studies regarding rutin (the main flavonoid from SE and present in important amounts for GE and CE) where it was included in phytosomes which penetrated in skin, higher amounts of phospholipids resulted in more viscous and adherent formulations with better skin permeability [58]. Additionally, it was formulated with ethosomes and evaluated for skin compatibility on volunteers (applying patches on the subjects’ backs for 48 h, three times a week), resulting in a harmless profile. According to permeability results in comparison to free rutin, it was noted that ethosomes penetrated through the *stratum corneum* while free rutin remained in the surface layers [59]. Other authors evaluated the penetration of rutin from ethanolic, aqueous, liposomal, and ethosomal formulations and revealed the following succession: ethosomes > ethanolic solution > liposomes > aqueous solution [60]. Besides rutin, quercetin is another flavonoid found in the analyzed extracts [16] (higher amounts for SE) [16]. Formulated as PEV, quercetin led to tissue regeneration by stimulating collagen fibers’ growth, restructuring the skin’s architecture. It was also incorporated in glycerosomal formulations (glycerol 10–50%) for a local antioxidant effect, and as a liposomal formulation was found inside keratinocytes (in vitro) [61]. As a perspective of the present study, further research will aim towards the analysis of the obtained nanoformulations in interaction with skin structures and the evaluation of their pharmacokinetic and pharmacodynamic profiles.

## 5. Conclusions

This research resulted in obtaining nine nanoformulations using three vegetal extracts (SE—*Sophorae flos extract*, CE—*Calendulae flos* extract, and GE—*Ginkgo bilobae folium* extract) and two biopolymers (PHB and PLGA). Their spreadability was evaluated, with the best results for CE-PHB, and also their pH, with values of 5.5 for all samples. Advanced physicochemical methods of analysis were performed for characterization. ATR-FTIR and XRD were applied to compare the nanoformulations with their base components. ATR-FTIR results highlighted the main bands for the stretching vibrations of O-H (more pronounced), C=O, and C-H (asymmetric and symmetric for methyl and methylene). The bands of the nanoformulations were in the same regions as their base components, with some noted differences in wavenumber and absorbance, suggesting interactions and confirming the formation of complexes or adducts. XRD analysis revealed that the prepared nanoformulations were in an amorphous phase, an important factor of drug delivery properties which can be the aim of further studies. Morphology and roughness were investigated by AFM, and some differences were observed: SE-PHB appeared more compact and uniform compared to CE-PHB and GE-PHB; SE-PLGA revealed a mixture of large nanostructures (~500 nm) and small nanoparticles; and SE-PHB-PLGA exhibited aggregative phenomena and small nanoparticles; while CE-PLGA, GE-PLGA, CE-PHB-PLGA, and GE-PHB-PLGA showed only small nanoparticles. Their roughness varied depending on the extract in the following order: CE-polymer > SE-polymer > GE-polymer. AFM confirmed the formation of nanoparticles which can be further subjected to optimization processes. Moreover, thermogravimetric analysis was performed to estimate the possibility of impregnation in a compression stocking matrix, noting variation in the order of CE > SE > GE, with the best results for CE-PHB. More detailed analysis of the material impregnation, and also the assessment of the biopharmaceutical, pharmacokinetic, and pharmacodynamic properties of these nanoformulations are considered for research perspectives.

## Figures and Tables

**Table 1:** Spreadability assay results.

	Weight (g)						
	150	250	350	450	550	650	750
Sample	The Spreading Surface * (cm^2^)						
SE-PHB	58.54 ± 3.22	92.18 ± 5.17	102.13 ± 8.25	110.56 ± 3.89	114.31 ± 2.19	116.84 ± 1.92	117.49 ± 2.21
CE-PHB	59.99 ± 6.40	99.11 ± 5.37	109.97 ± 5.56	125.34 ± 6.04	136.85 ± 7.51	141.00 ± 6.31	141.72 ± 7.24
GE-PHB	56.28 ± 2.02	91.79 ± 11.04	108.10 ± 4.65	121.36 ± 2.99	130.64 ± 2.03	136.78 ± 2.07	137.48 ± 2.39
SE-PLGA	42.61 ± 1.76	47.76 ± 1.22	55.41 ± 2.30	61.73 ± 2.12	67.93 ± 3.89	70.36 ± 1.72	73.36 ± 0.87
CE-PLGA	54.54 ± 2.71	73.93 ± 5.53	82.81 ± 5.65	91.58 ± 3.39	99.70 ± 5.64	105.69 ± 6.25	109.34 ± 4.87
GE-PLGA	59.44 ± 2.73	69.90 ± 4.50	78.52 ± 3.14	86.06 ± 5.76	95.68 ± 8.11	103.93 ± 8.22	105.16 ± 9.24
SE-PHB-PLGA	51.52 ± 2.54	59.88 ± 1.58	68.39 ± 2.24	75.40 ± 1.54	82.23 ± 3.33	88.27 ± 5.96	92.70 ± 0.98
CE-PHB-PLGA	63.60 ± 2.46	87.11 ± 2.53	93.85 ± 2.63	103.23 ± 2.74	108.70 ± 2.82	116.23 ± 4.80	117.50 ± 3.98
GE-PHB-PLGA	52.38 ± 2.68	70.36 ± 1.72	81.15 ± 2.43	87.66 ± 2.53	95.57 ± 2.65	99.07 ± 2.70	100.86 ± 4.49

* mean of three determinations ± standard deviation; SE = *Sophorae flos* extract, GE = *Ginkgo bilobae folium* extract; CE = *Calendulae flos* extract; PHB = polyhydroxybutyrate, PLGA = polylactic-co-glycolic acid.

**Table 2:** Roughness parameters for PHB formulations; Rpv = peak to valley height; Rq = root mean square roughness; Ra = average roughness; SE = *Sophorae flos* extract; CE = *Calendulae flos* extract; GE = *Ginkgo bilobae folium* extract.

Area	Whole Surface						Red Line Profile					
Scale	8 × 8 μm^2^			2 × 2 μm^2^			8 μm^2^			2 μm^2^		
Parameter (nm)	Rpv	Rq	Ra	Rpv	Rq	Ra	Rpv	Rq	Ra	Rpv	Rq	Ra
SE-PHB	170.768	16.443	11.744	27.010	3.286	2.585	91.065	20.301	13.954	18.289	3.395	2.765
CE-PHB	224.990	29.470	21.029	46.588	5.047	3.955	166.909	42.605	32.942	23.874	5.161	4.394
GE-PHB	113.708	11.175	8.689	10.901	1.155	0.943	53.389	14.300	11.886	5.201	1.152	0.869

**Table 3:** Roughness parameters for PLGA formulations (Rpv = peak to valley height; Rq = root mean square roughness; Ra = average roughness, SE = *Sophorae flos* extract, CE = *Calendulae flos* extract, GE = *Ginkgo bilobae folium* extract).

Area	Whole Surface						Red Line Profile					
Scale	8 × 8 μm^2^			2 × 2 μm^2^			8 μm^2^			2 μm^2^		
Parameter (nm)	Rpv	Rq	Ra	Rpv	Rq	Ra	Rpv	Rq	Ra	Rpv	Rq	Ra
SE-PLGA	166.137	31.919	27.101	45.421	7.394	5.963	124.934	32.250	27.486	14.222	3.948	3.333
CE-PLGA	175.082	12.202	8.726	19.523	2.184	1.710	61.694	9.614	6.917	9.820	1.837	1.442
GE-PLGA	94.713	14.761	10.703	18.334	2.633	2.125	42.459	10.898	9.441	11.738	2.506	1.931

**Table 4:** Roughness parameters for PHB-PLGA formulations (Rpv = peak to valley height; Rq = root mean square roughness; Ra = average roughness, SE = *Sophorae flos* extract, CE = *Calendulae flos* extract, GE = *Ginkgo bilobae folium* extract).

Area	Whole Surface						Red Line Profile					
Scale	8 × 8 μm^2^			2 × 2 μm^2^			8 μm^2^			2 μm^2^		
Parameter (nm)	Rpv	Rq	Ra	Rpv	Rq	Ra	Rpv	Rq	Ra	Rpv	Rq	Ra
SE-PHB-PLGA	249.559	38.634	27.655	86.305	9.802	7.801	226.326	68.959	57.453	81.669	15.593	11.810
CE-PHB-PLGA	191.320	19.981	15.339	19.386	2.055	1.568	33.433	8.625	7.514	8.011	1.767	1.421
GE-PHB-PLGA	114.624	17.087	14.496	30.014	2.359	1.807	72.339	18.340	15.774	9.472	2.307	1.967

**Table 5:** Sample content estimated by thermogravimetric assay: SE = *Sophorae flos* extract, CE = *Calendulae flos* extract, GE = *Ginkgo bilobae folium* extract, PHB = polyhydroxybutyrate, PLGA = polylactic-co-glycolic acid, M = compression stockings matrix.

Sample	Formulation (%wt.)	Matrix (%wt.)
M-SE-PHB	7.8	92.2
M-CE-PHB	9.5	90.5
M-GE-PHB	7.0	93.0
M-SE-PLGA	5.4	94.6
M-CE-PLGA	8.1	91.9
M-GE-PLGA	5.2	94.8
M-SE-PHB-PLGA	8.5	91.5
M-CE-PHB-PLGA	6.7	93.3
M-GE-PHB-PLGA	4.9	95.1

## Data Availability

Data are contained within the article and Supplementary Materials.

## Supplementary Materials

The following supporting information can be downloaded at https://www.mdpi.com/article/10.3390/polym16101362/s1: Figure S1: Spreadability variation depending on type of extract and biopolymer. Figure S2: ATR-FTIR spectrum of polyhydroxybutyrate (PHB). Figure S3: ATR-FTIR spectrum of polylactic-co-glycolic acid (PLGA). Figure S4: ATR-FTIR spectrum of *Sophorae flos extract* (SE). Figure S5: ATR-FTIR spectrum of *Ginkgo bilobae folium* extract (GE). Figure S6: ATR-FTIR spectrum of *Calendulae flos* extract (CE). Figure S7: ATR-FTIR spectrum of SE-PHB formulation. Figure S8: ATR-FTIR spectrum of GE-PHB formulation. Figure S9: ATR-FTIR spectrum of CE-PHB formulation. Figure S10: ATR-FTIR spectrum of SE-PLGA formulation. Figure S11: ATR-FTIR spectrum of GE-PLGA formulation. Figure S12: ATR-FTIR spectrum of CE-PLGA formulation. Figure S13. ATR-FTIR spectrum of SE-PHB-PLGA formulation. Figure S14: ATR-FTIR spectrum of GE-PHB-PLGA formulation. Figure S15: ATR-FTIR spectrum of CE-PHB-PLGA formulation. Figure S16: ATR-FTIR spectra of vegetal extracts and biopolymers. Figure S17: ATR-FTIR spectrum of SE-PHB formulation in comparison with base components’ spectra. Figure S18: ATR-FTIR spectrum of GE-PHB formulation in comparison with base components’ spectra. Figure S19: ATR-FTIR spectrum of CE-PHB formulation in comparison with base components’ spectra. Figure S20: ATR-FTIR spectrum of SE-PLGA formulation in comparison with base components’ spectra. Figure S21: ATR-FTIR spectrum of GE-PLGA formulation in comparison with base components’ spectra. Figure S22: ATR-FTIR spectrum of CE-PLGA formulation in comparison with base components’ spectra. Figure S23: ATR-FTIR spectrum of SE-PHB-PLGA formulation in comparison with base components’ spectra. Figure S24: ATR-FTIR spectrum of GE-PHB-PLGA formulation in comparison with base components’ spectra. Figure S25: ATR-FTIR spectrum of CE-PHB-PLGA formulation in comparison with base components’ spectra. Figure S26: Diffraction patterns of formulations in comparison with base components. Figure S27: 3D AFM images of PHB formulation samples. Figure S28: 3D AFM images of PLGA formulation samples. Figure S29: 3D AFM images of PHB-PLGA formulation samples. Table S1: ATR-FTIR analysis results—principal peaks’ description.
