# Supplementary material for: Preparation and Preliminary Analysis of Several Nanoformulations Based on Plant Extracts and Biodegradable Polymers as a Possible Application for Chronic Venous Disease Therapy
Source: Polymers (Basel). 2024 May 10;16(10):1362. doi: 10.3390/polym16101362 (PMC11125073; doi:10.3390/polym16101362)
Supplement: Supplementary file 1 [file polymers-16-01362-s001.zip › polymers-2986627-supplementary.pdf]

Article

Preparation and Preliminary Analysis of Several Nanoformulations Based on Plant Extracts and Biodegradable Polymers as a Possible Application for Chronic Venous Disease Therapy

Supplementary Materials

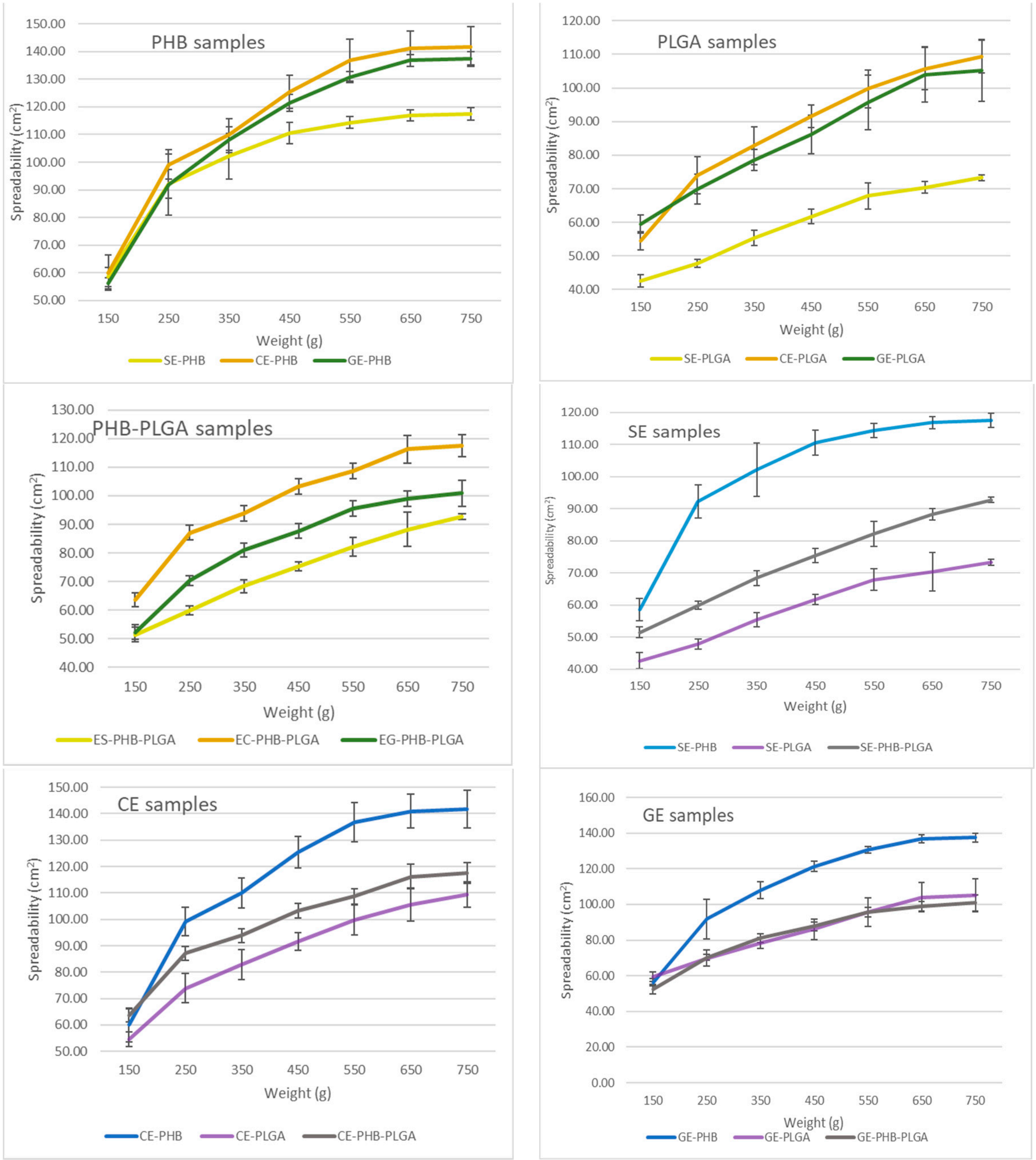

Figure S1. Spreadability variation depending on type of extract and biopolymer. SE = *Sophorae flos* extract, GE = *Ginkgo bilobae folium* extract; CE = *Calendulae flos* extract; PHB = polyhydroxybutyrate, PLGA = polylactic-co-glycolic acid.

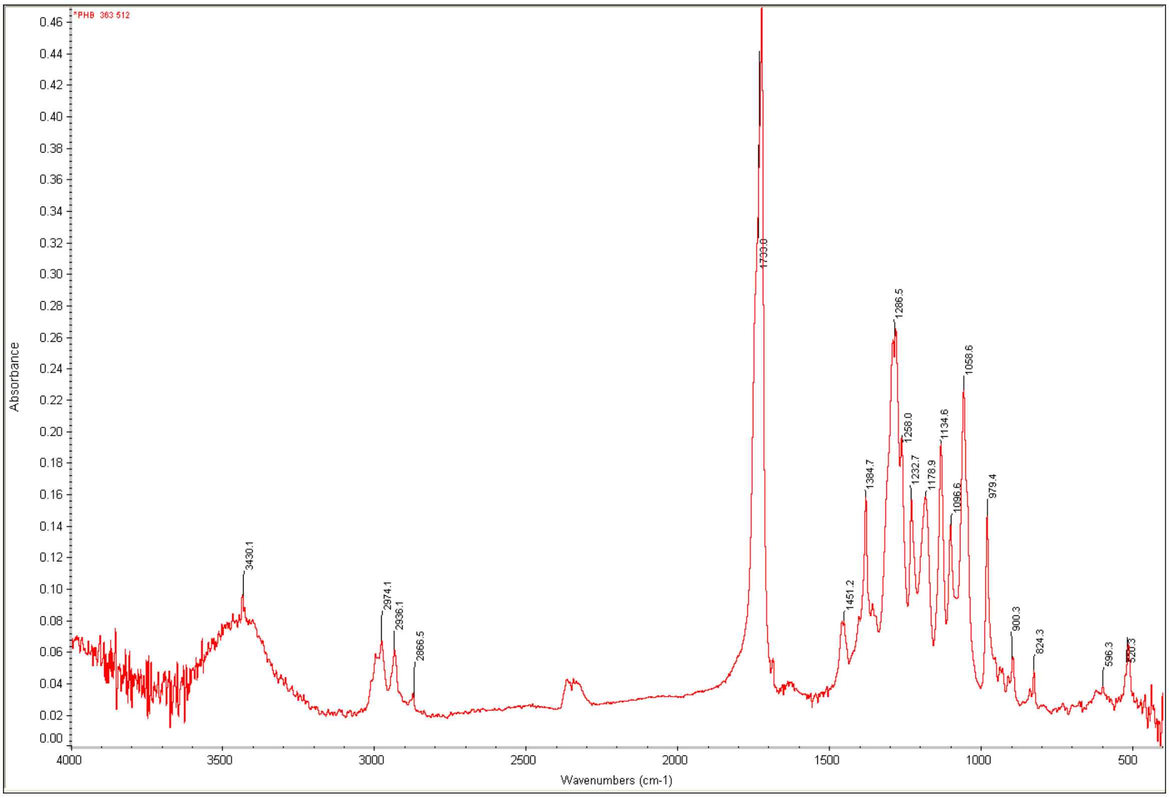

Figure S2. ATR-FTIR spectrum of polyhydroxybutyrate (PHB).

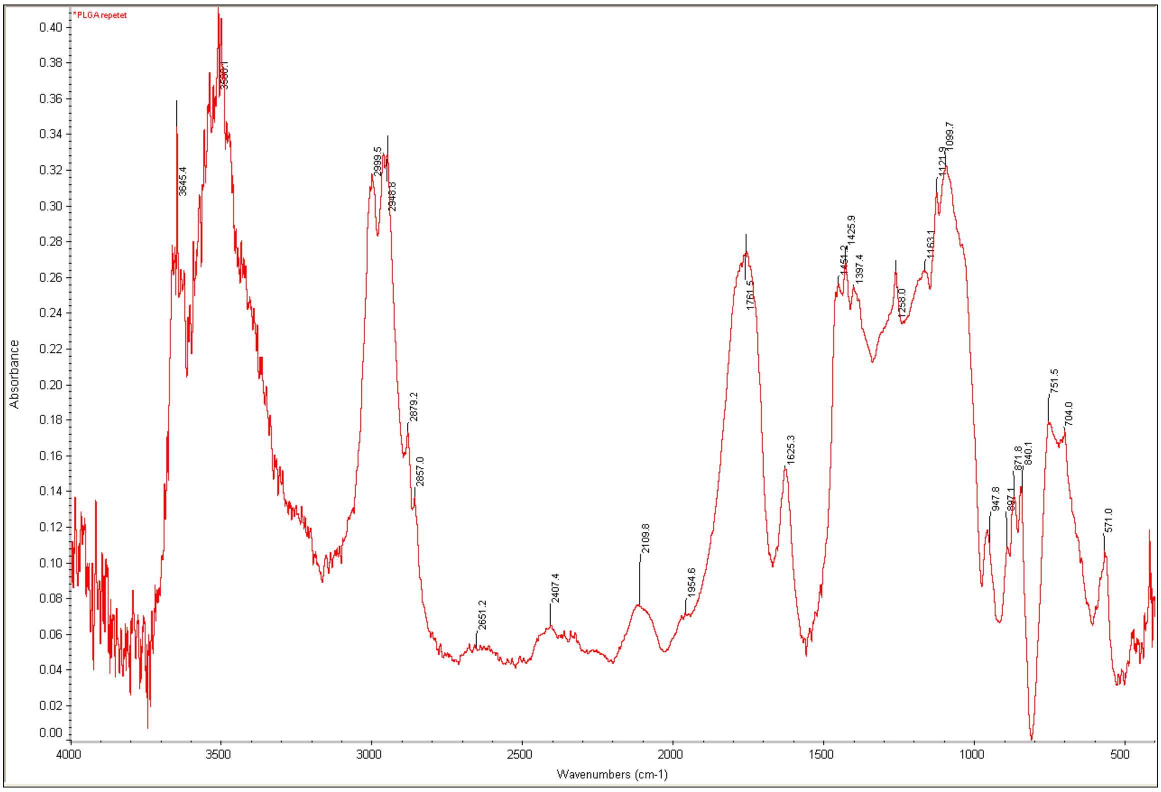

Figure S3. ATR-FTIR spectrum of polylactic-co-glycolic acid (PLGA).

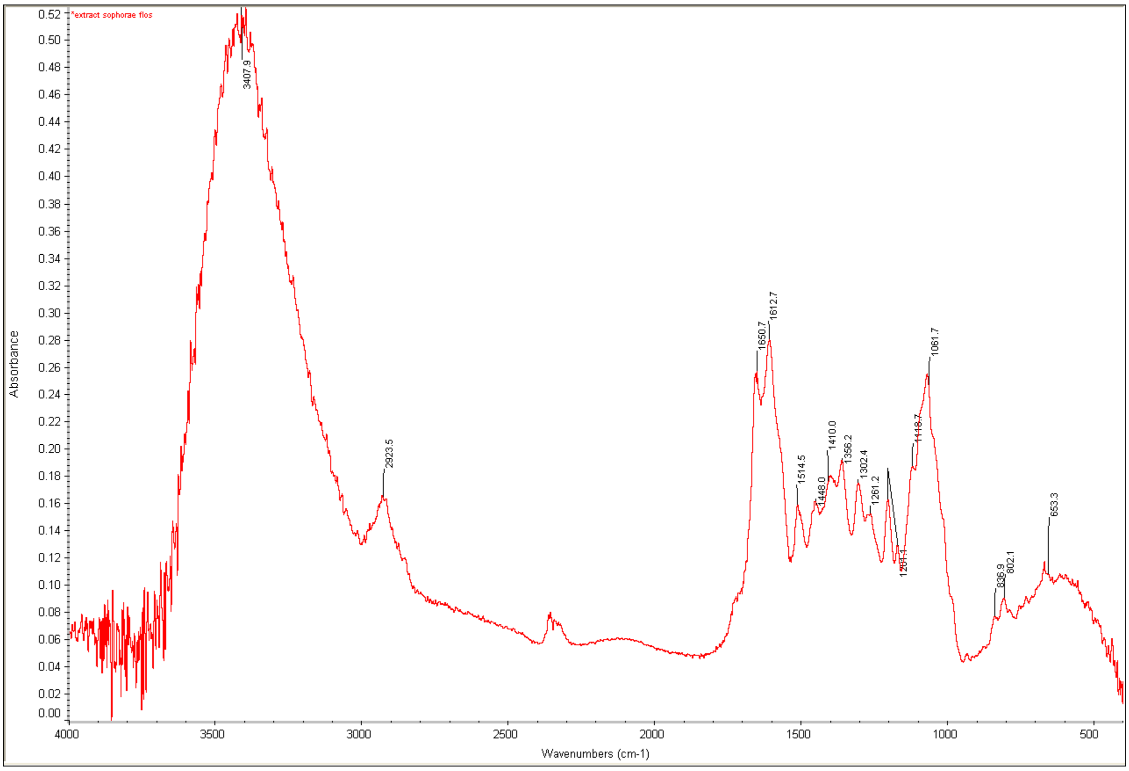

Figure S4. ATR-FTIR spectrum of *Sophorae flos* extract (SE).

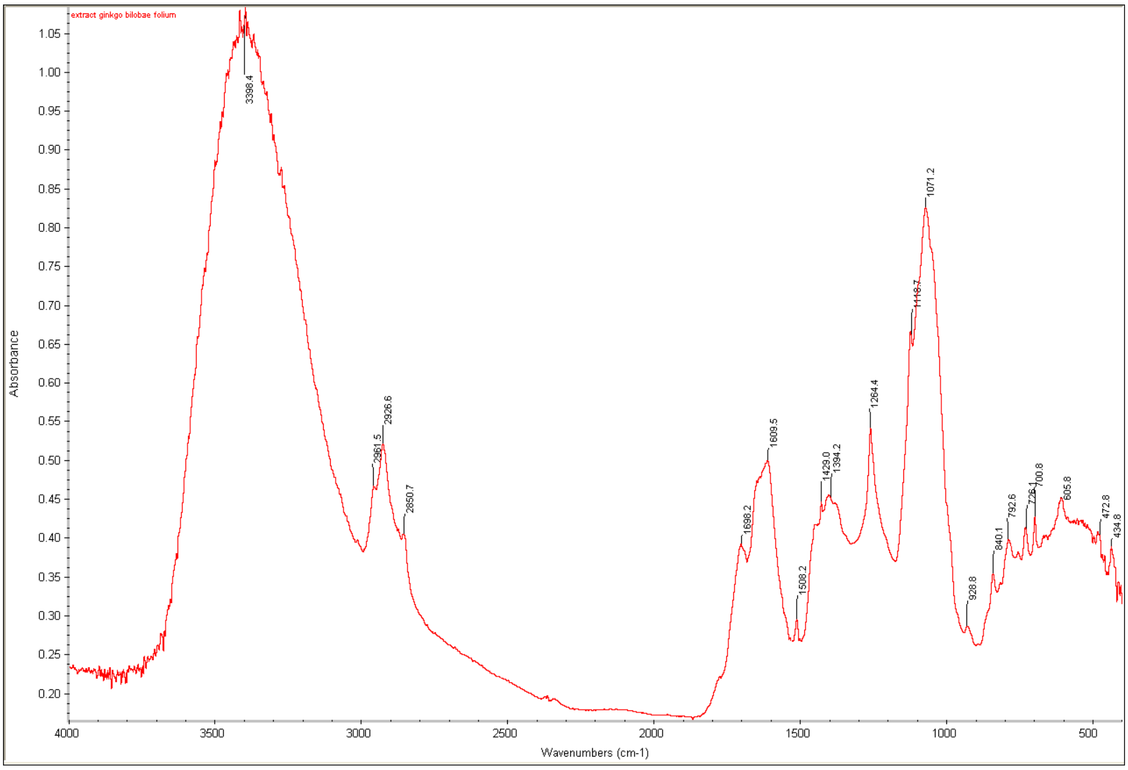

Figure S5. ATR-FTIR spectrum of *Ginkgo bilobae folium* extract (GE).

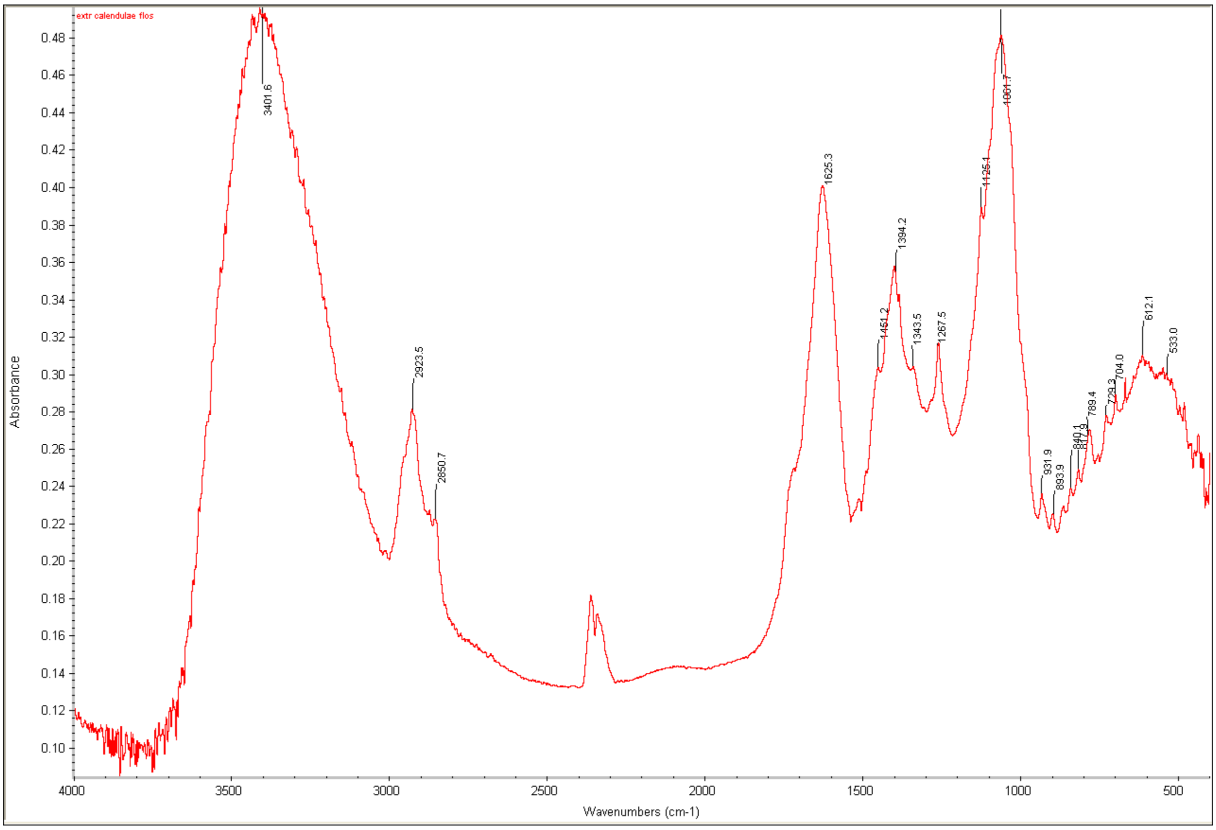

Figure S6. ATR-FTIR spectrum of *Calendulae flos* extract (CE).

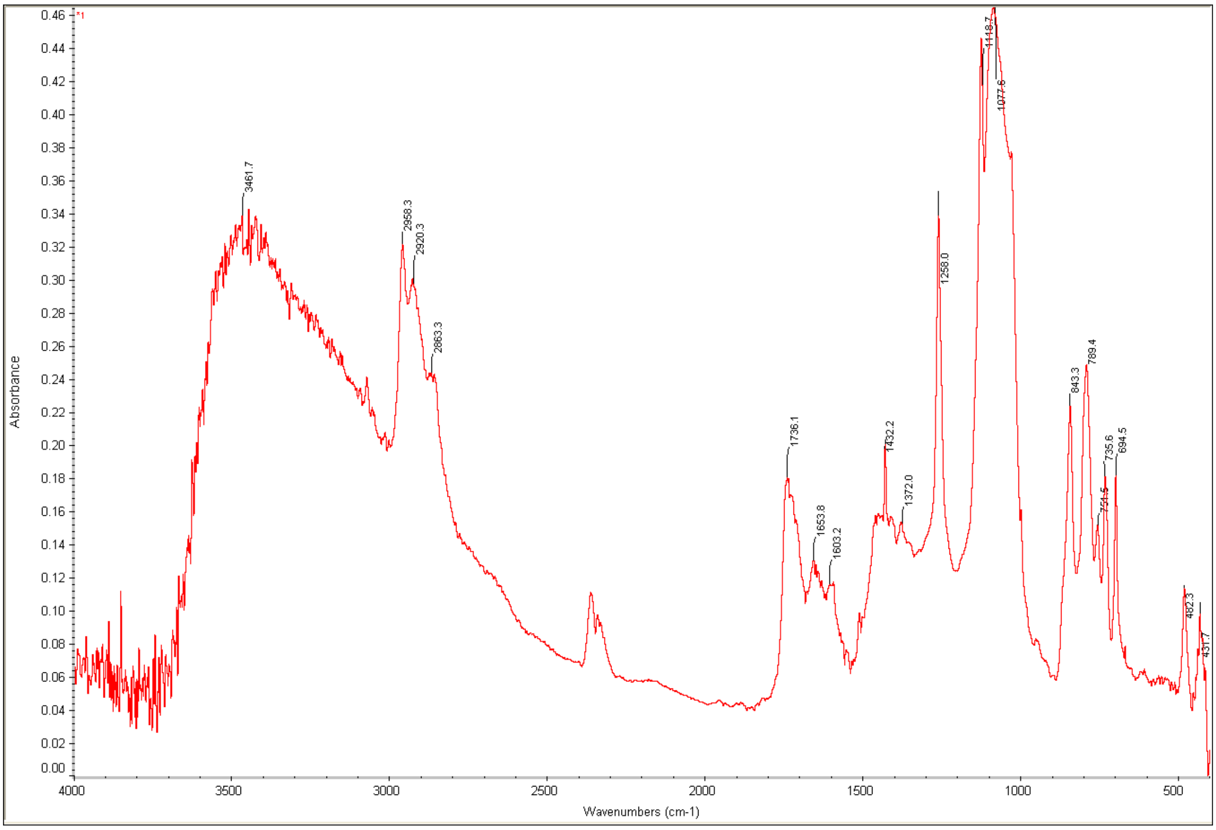

Figure S7. ATR-FTIR spectrum of SE-PHB formulation.

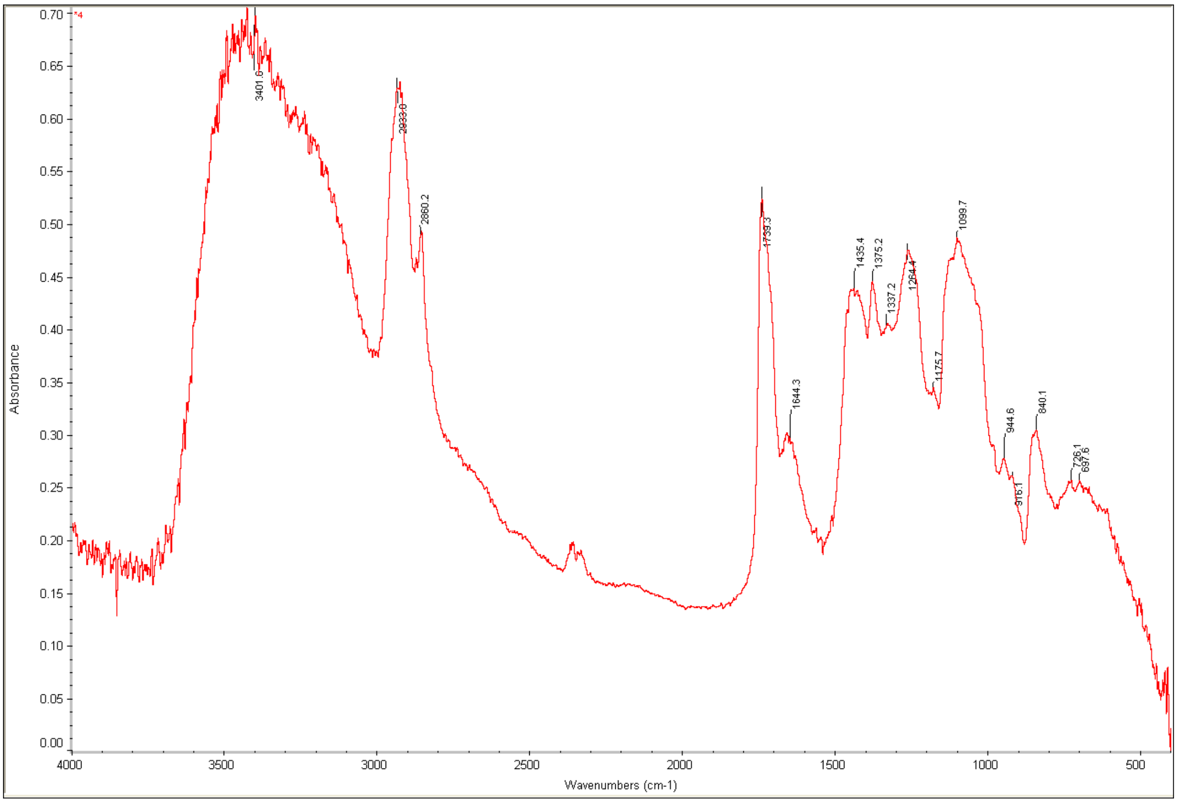

Figure S8. ATR-FTIR spectrum of GE-PHB formulation.

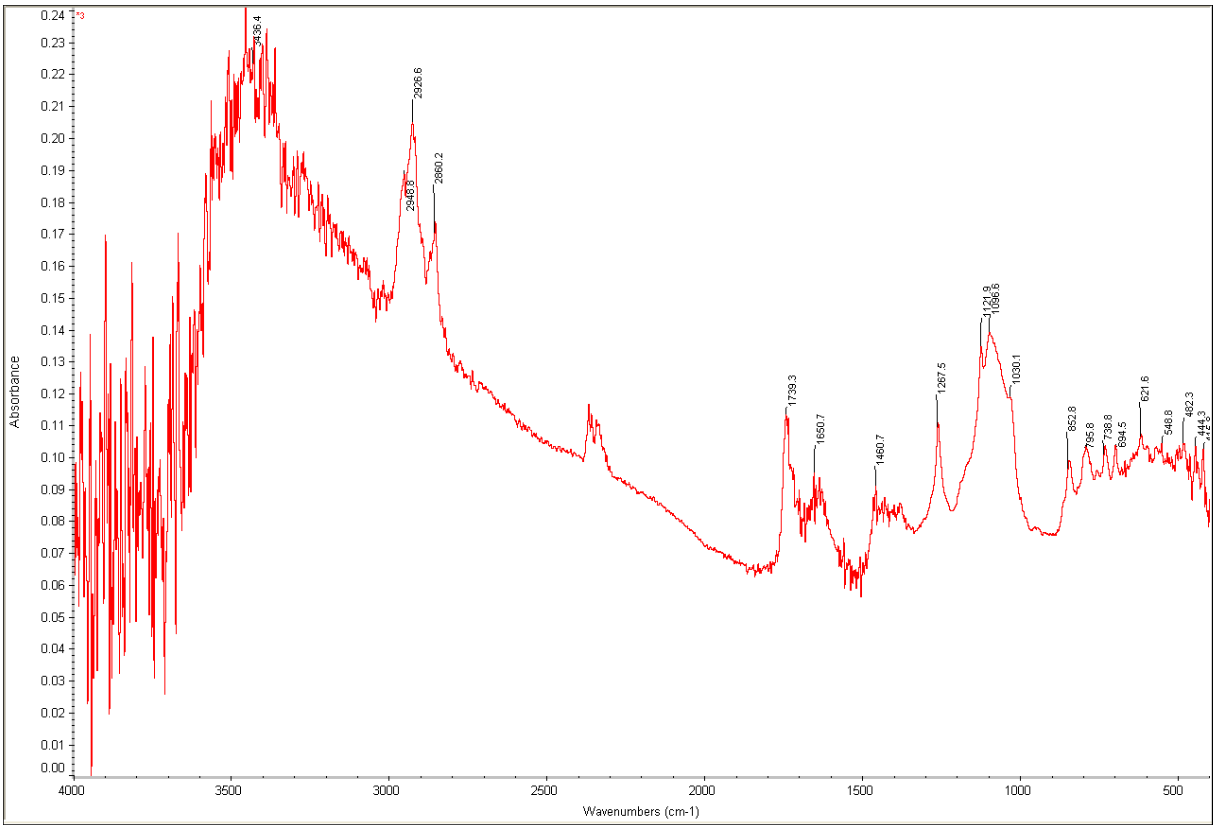

Figure S9. ATR-FTIR spectrum of CE-PHB formulation.

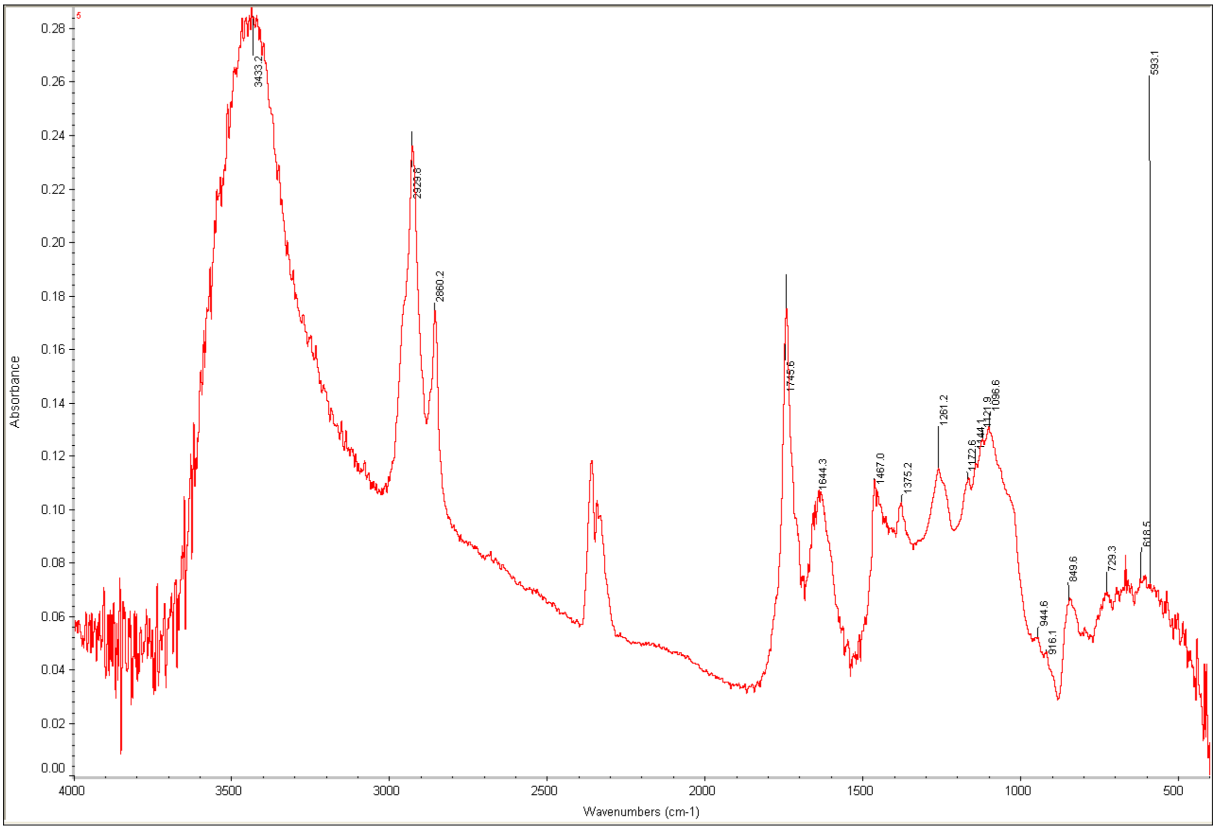

Figure S10. ATR-FTIR spectrum of SE-PLGA formulation.

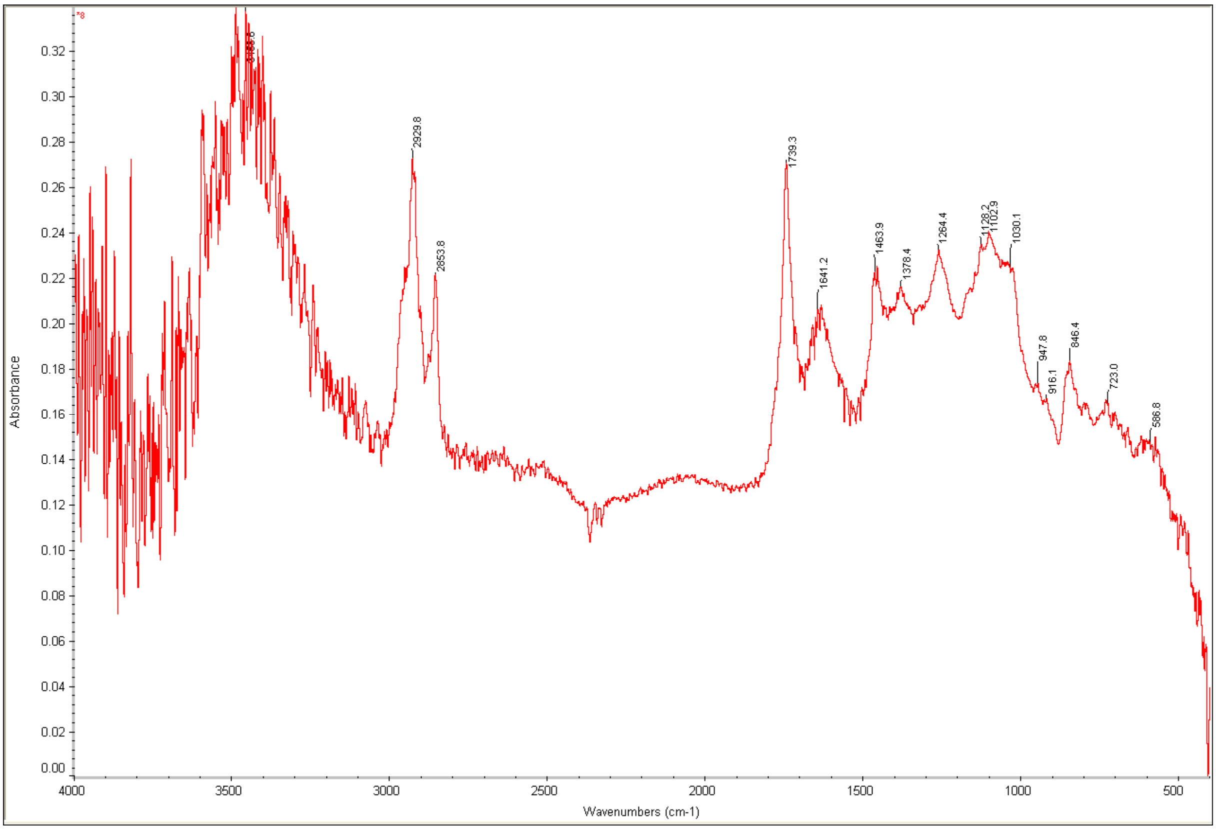

Figure S11. ATR-FTIR spectrum of GE-PLGA formulation.

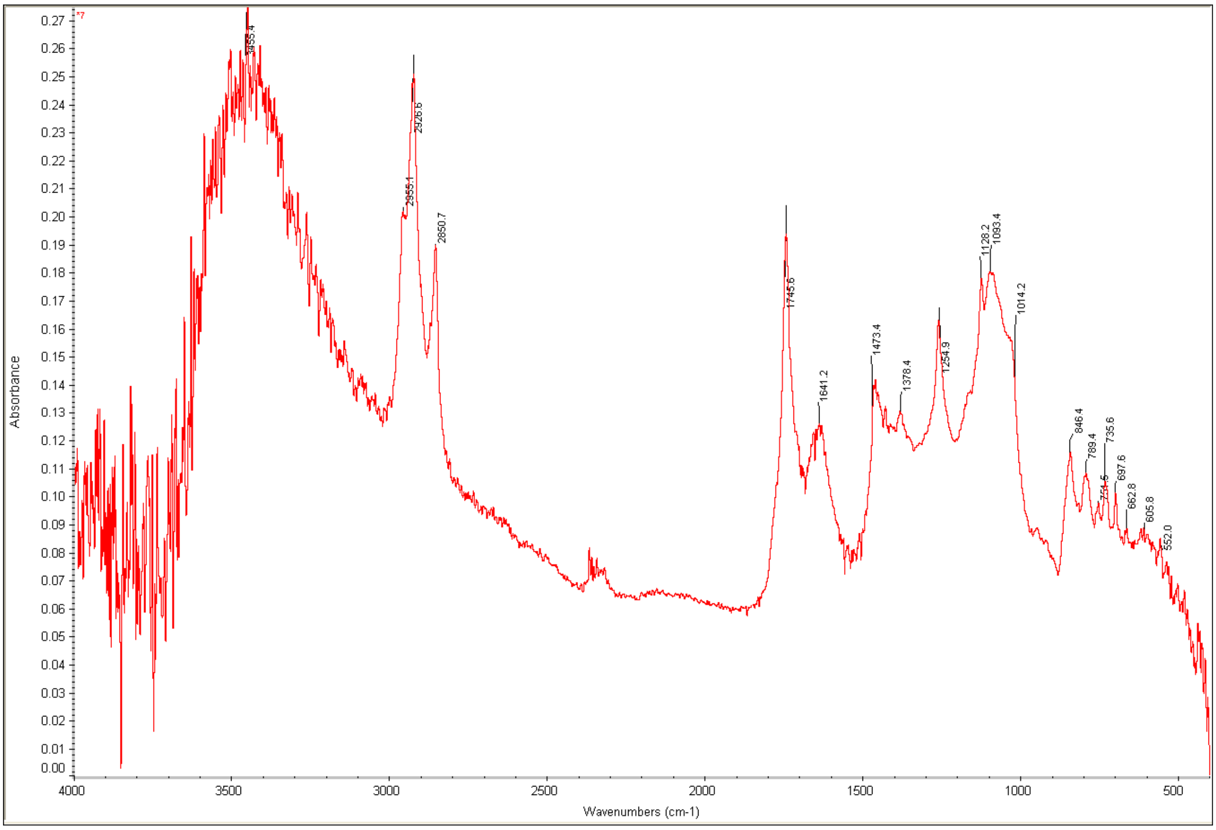

Figure S12. ATR-FTIR spectrum of CE-PLGA formulation.

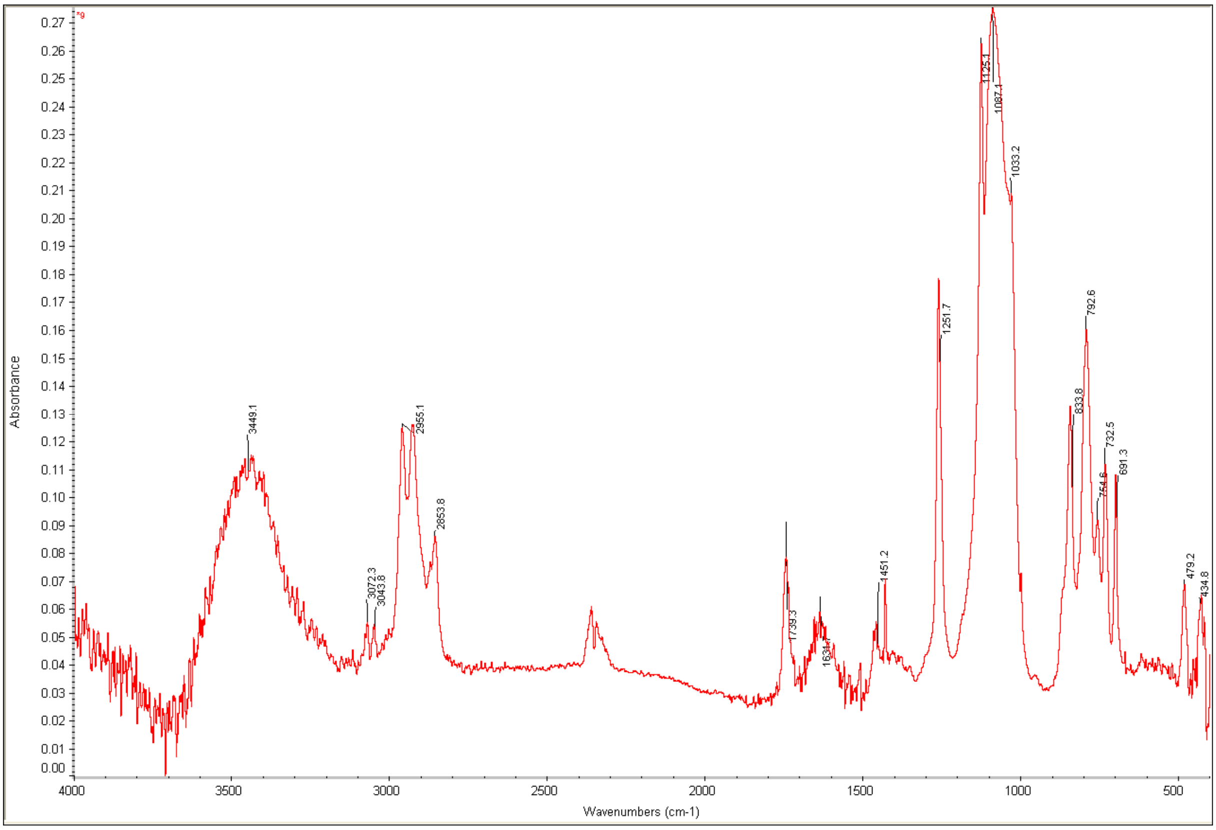

Figure S13. ATR-FTIR spectrum of SE-PHB-PLGA formulation.

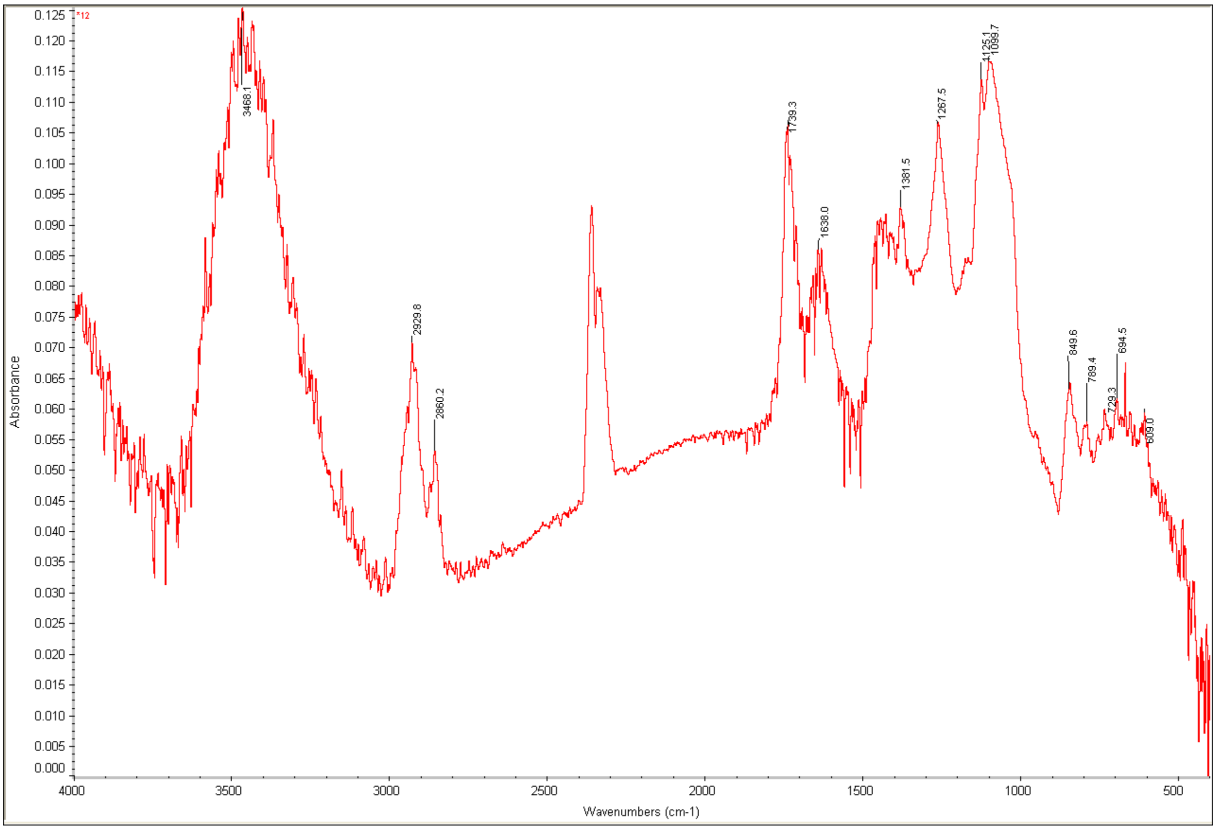

Figure S14. ATR-FTIR spectrum of GE-PHB-PLGA formulation.

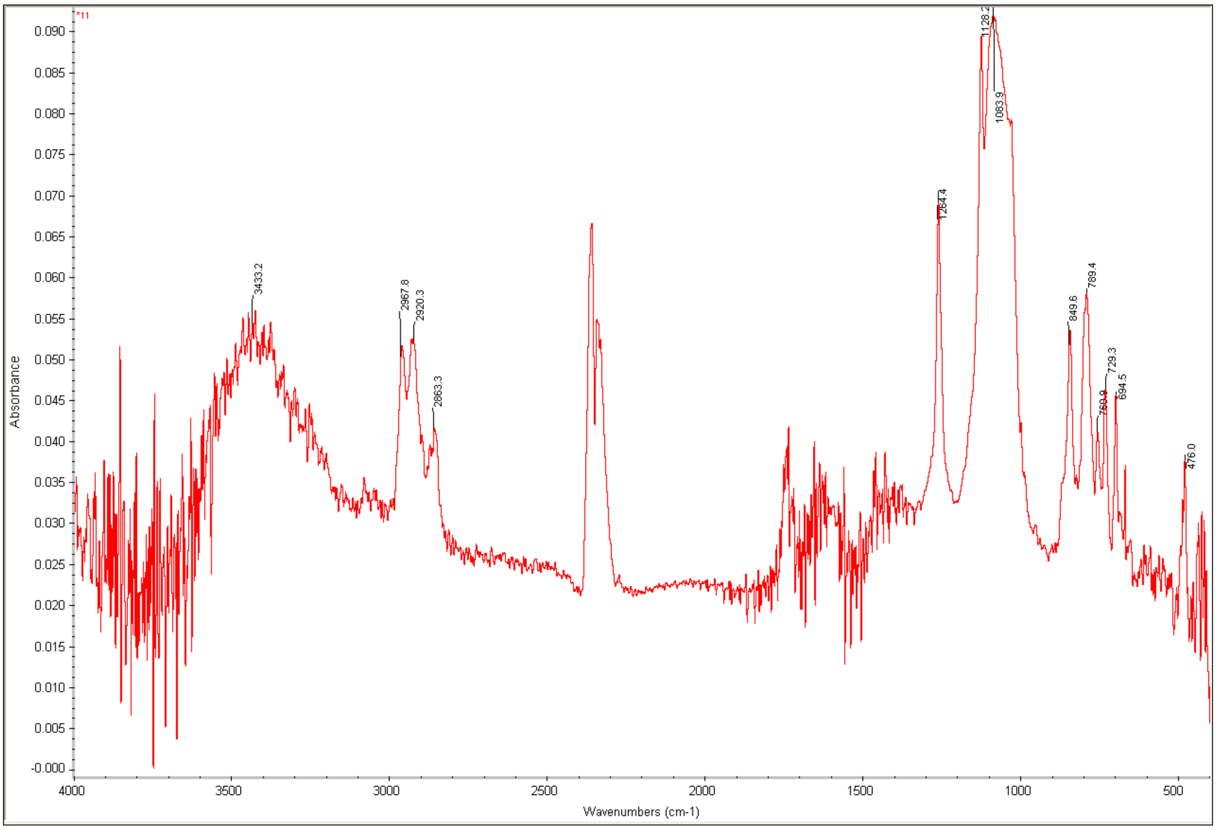

Figure S15. ATR-FTIR spectrum of CE-PHB-PLGA formulation.

Table S1. ATR-FTIR Analysis results—principal peaks' description.

Spectral region	Base components	Formulations	Peak description
4000-2500 cm^{-1} (single bonds region)	3645.4 (PLGA)<br>3500.1 (PLGA)<br>3430.1 (PHB)<br>3407.9 (SE)<br>3401.6 (CE)<br>3398.4 (GE)	3461.7 (SE-PHB)<br>3401.6 (GE-PHB)<br>3436.4 (CE-PHB)<br>3488.8 (GE-PLGA)<br>3455.4 (CE-PLGA)<br>3433.2 (SE-PLGA)<br>3449.1 (SE-PHB-PLGA)<br>3072.3 (SE-PHB-PLGA)<br>3043.8 (SE-PHB-PLGA)<br>3468.1 (GE-PHB-PLGA)<br>3433.2 (CE-PHB-PLGA)	>3000 stretching vibrations of O-H bonds from alcohols and phenols<br>3550-3450 cm^{-1} stretching vibrations of dimeric O-H
	2963.1 (PHB)<br>2961.5 (GE)	2958.3 (SE-PHB)<br>2955.1 (CE-PLGA, SE-PHB-PLGA)<br>2967.8 (CE-PHB-PLGA)	2970-2950 cm^{-1} asymmetric stretching vibrations of C-H bonds from methyl groups
	2926.6 (GE)<br>2923.5 (SE, CE)	2920.3 (SE-PHB, CE-PHB-PLGA)<br>2933 (GE-PHB)<br>2926.6 (CE-PHB, CE-PLGA)<br>2929.8 (SE-PLGA, GE-PLGA, GE-PHB-PLGA)	2935-2915 cm^{-1} asymmetric stretching vibrations of C-H bonds from methylene groups
	2879.2 (PLGA)<br>2866.5 (PHB)	2860.2 (SE-PLGA)	2880-2860 cm^{-1} symmetric stretching vibrations of C-H bonds from methyl groups
	2857 (PLGA)<br>2850.7 (GE, CE)	2860.2 (SE-PLGA,GE-PHB)<br>2853.8 (GE-PLGA, SE-PHB-PLGA)<br>2850.7 (CE-PLGA)<br>2863.3 (CE-PHB-PLGA)	2865-2845 cm^{-1} symmetric stretching vibrations of C-H bonds from methylene groups
	2651.2 (PLGA)	2663.3 (SE-PHB)<br>2660.2 (CE-PHB, GE-PHB-PLGA)	Around 2660 cm^{-1} stretching vibrations of O-H bonds from acids groups
2000-1500 cm^{-1} (double bonds region)	1761.5 (PLGA)<br>1733 (PHB)<br>1698.2 (GE)	1736.1 (SE-PHB)<br>1745.6 (SE-PLGA, CE-PLGA)<br>1739.3 (GE-PHB, CE-PHB, GE-PLGA, SE-PHB-PLGA, GE-PHB-PLGA)	1780-1650 cm^{-1} stretching vibrations of C=O bonds and 1750-1725 cm^{-1} corresponding to C-O vibrations from ester groups
	1650.7 (SE)<br>1625.3 (CE, PLGA)<br>1612.7 (SE)	1653.8 (SE-PHB)<br>1644.3 (GE-PHB, SE-PLGA)<br>1650.7 (CE-PHB)<br>1631.7 (GE-PLGA, SE-PHB-PLGA)<br>1641.2 (CE-PLGA)<br>1638 (GE-PHB-PLGA)	1680-1620 cm^{-1} stretching vibrations of C=C bond from alkenil groups and 1650-1600 cm^{-1} corresponding to conjugated quinones or ketones
	1609.5 (GE)	1603.2 (SE-PHB)	Around 1600 cm^{-1} vibrations characteristic to C=C conjugated bonds
	1514.5 (SE)<br>1508.2 (GE)		Around 1500 cm^{-1} corresponding to aromatic rings

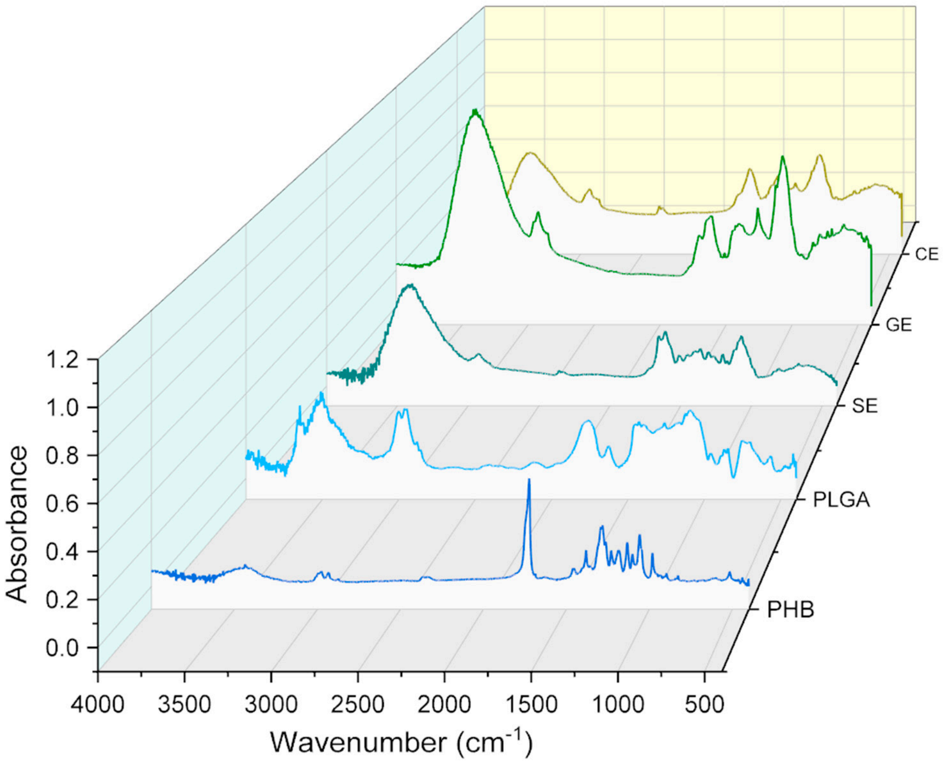

Figure S16. ATR-FTIR spectra of vegetal extracts and biopolymers.

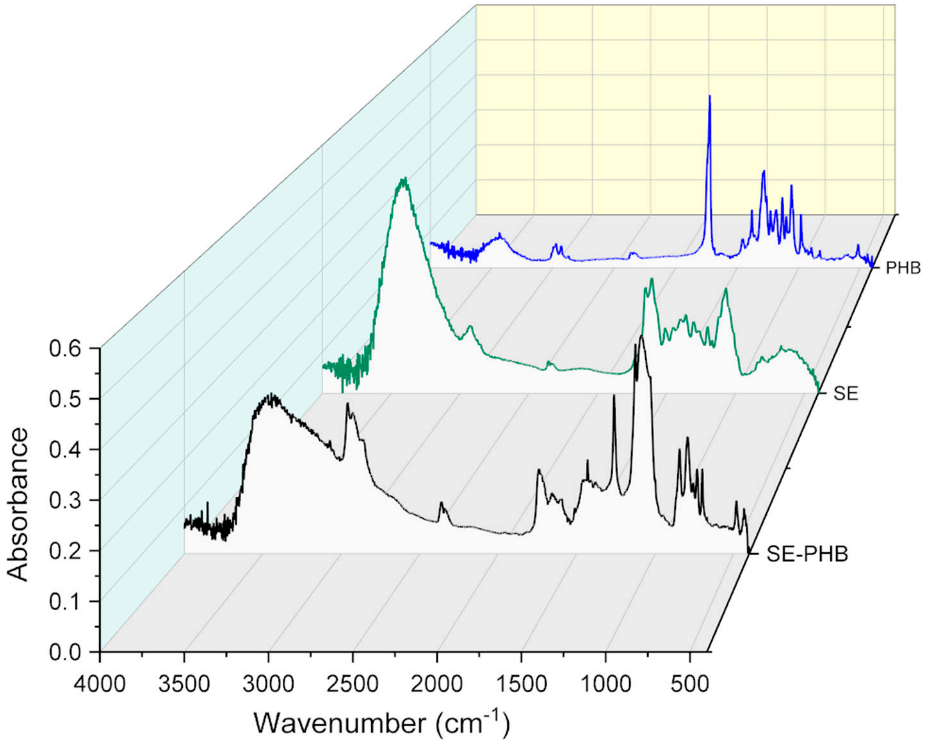

Figure S17. ATR-FTIR spectrum of SE-PHB formulation in comparison with base components' spectra.

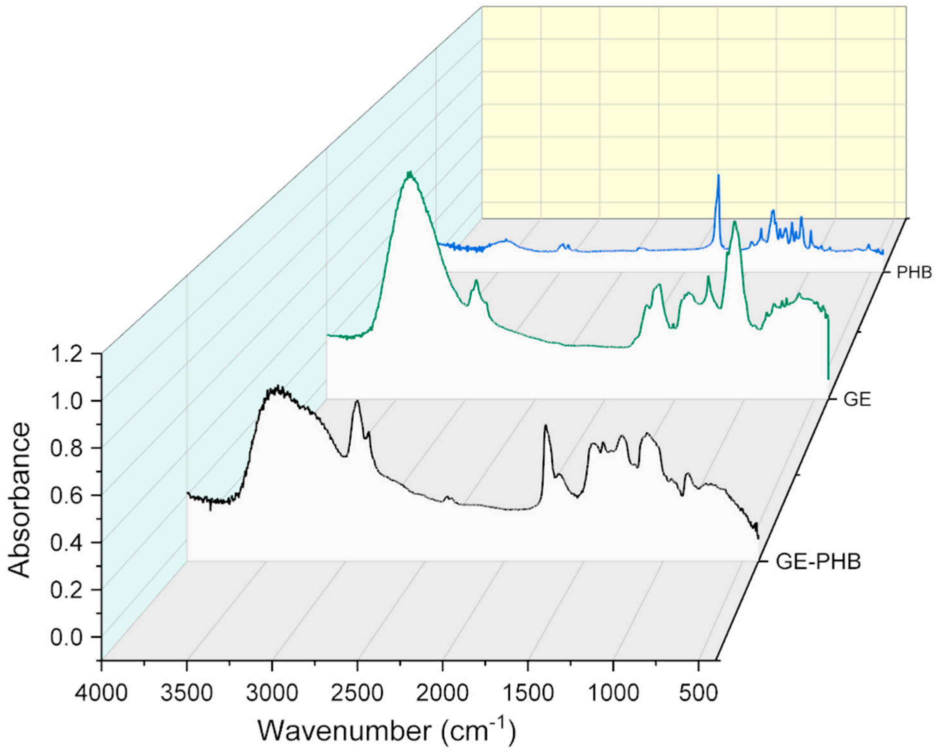

Figure S18. ATR-FTIR spectrum of GE-PHB formulation in comparison with base components' spectra.

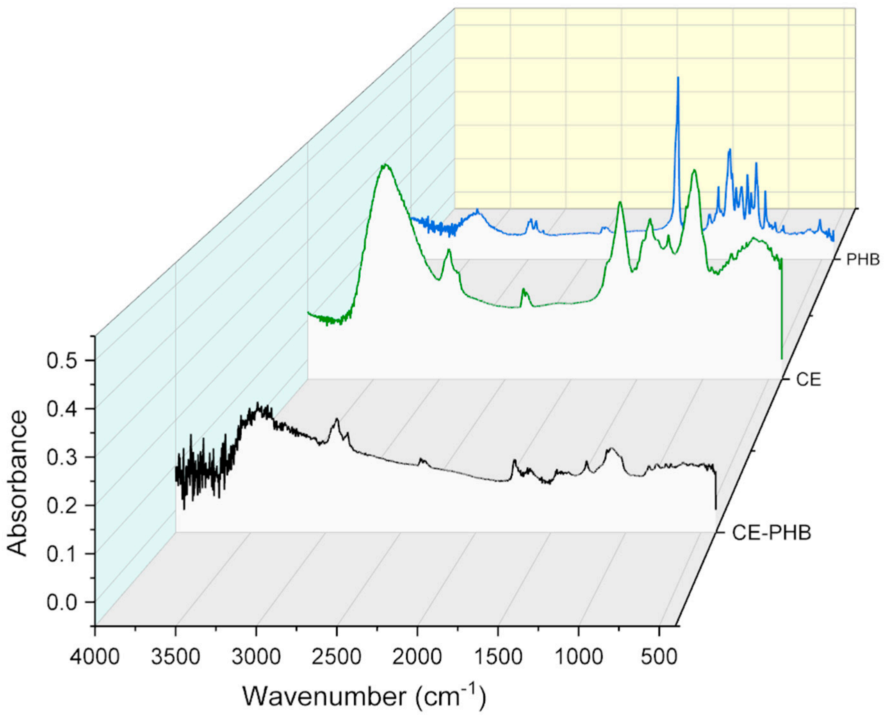

Figure S19. ATR-FTIR spectrum of CE-PHB formulation in comparison with base components' spectra.

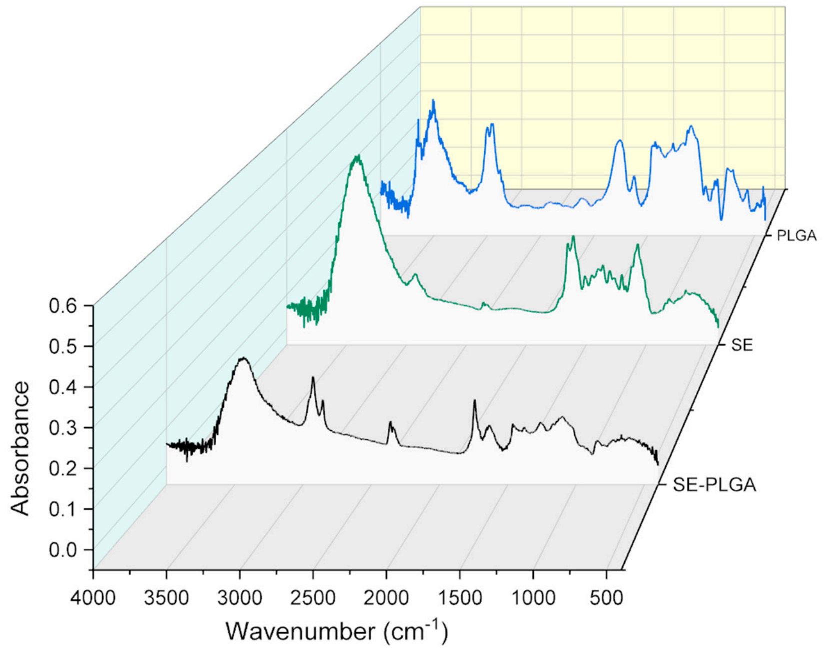

Figure S20. ATR-FTIR spectrum of SE-PLGA formulation in comparison with base components' spectra.

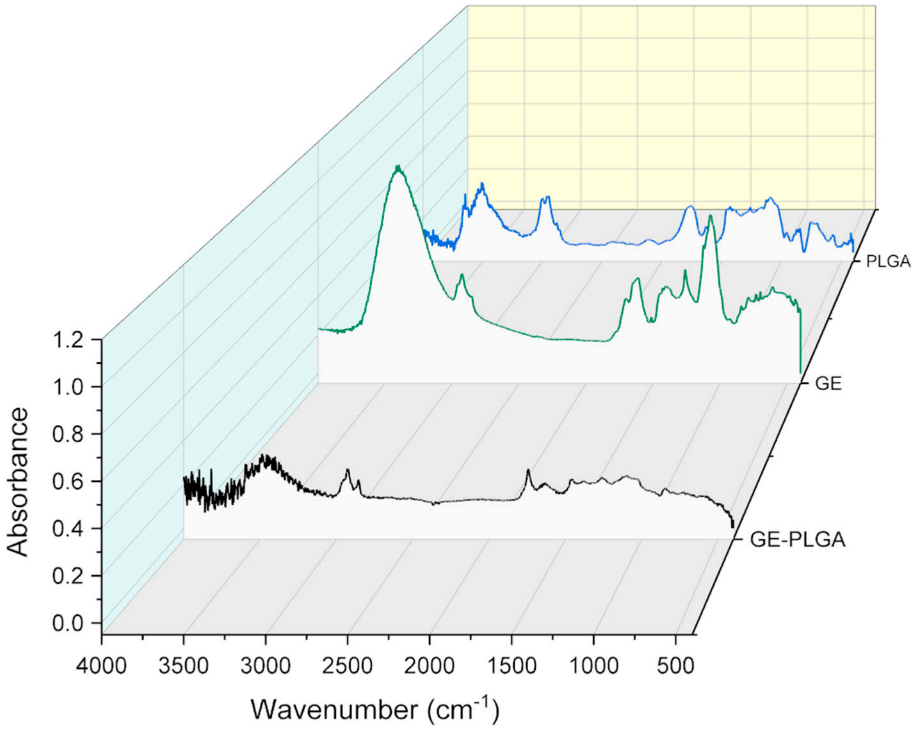

Figure S21. ATR-FTIR spectrum of GE-PLGA formulation in comparison with base components' spectra.

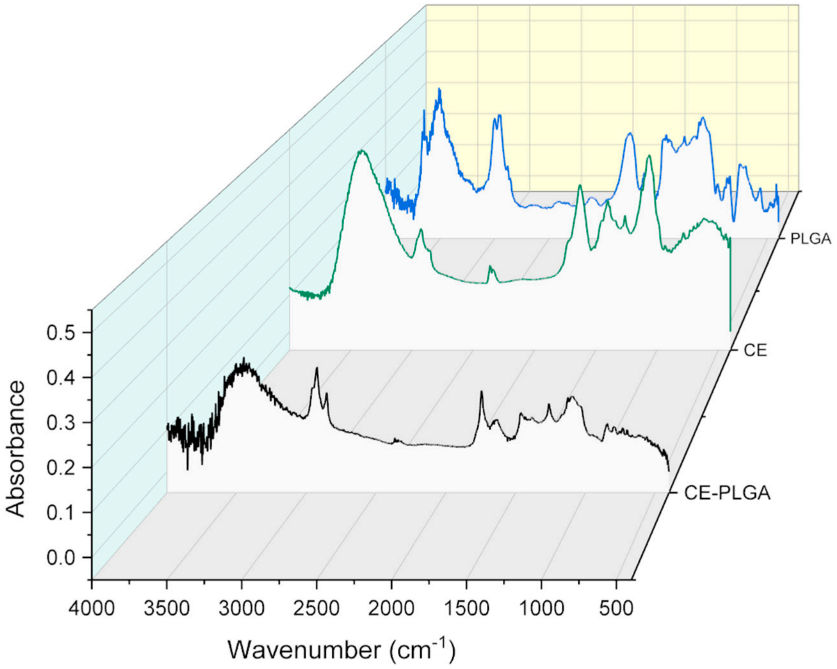

Figure S22. ATR-FTIR spectrum of CE-PLGA formulation in comparison with base components' spectra.

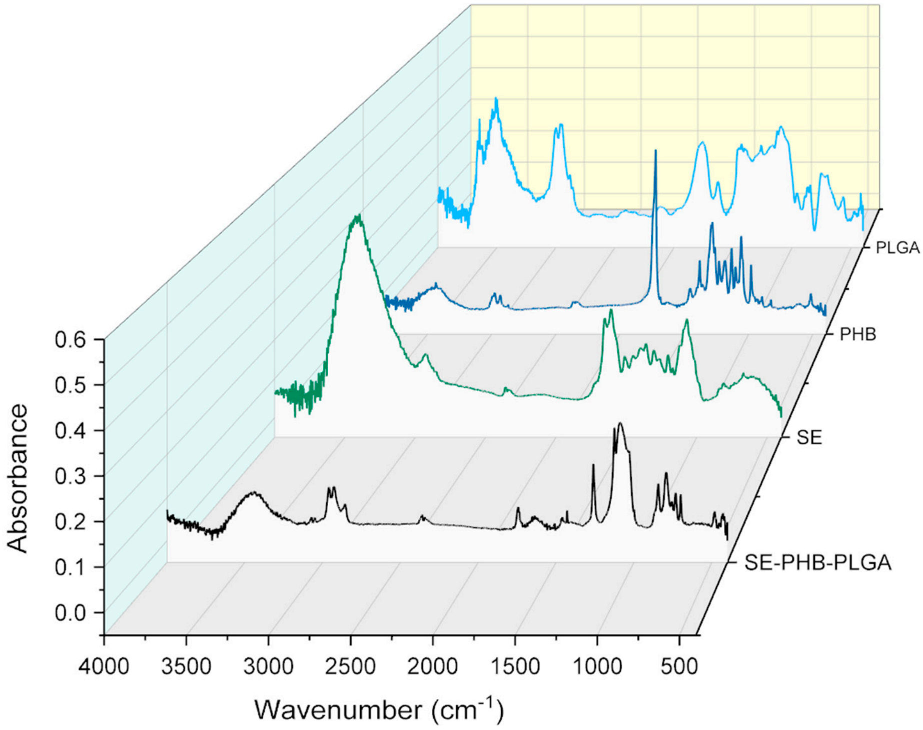

Figure S23. ATR-FTIR spectrum of SE-PHB-PLGA formulation in comparison with base components' spectra.

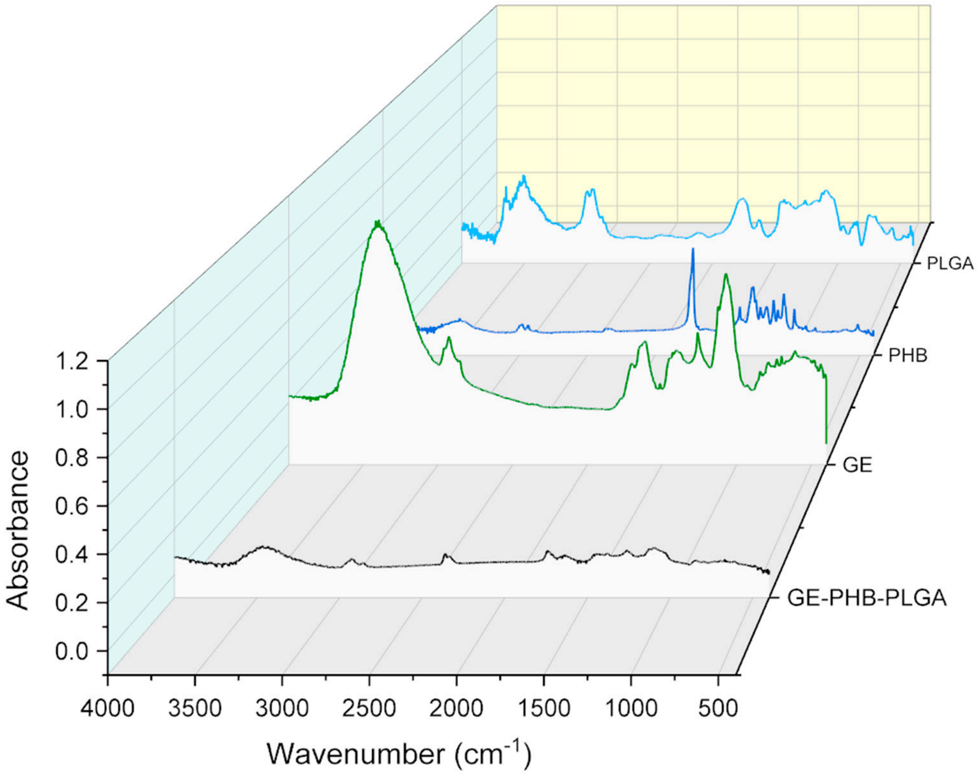

Figure S24. ATR-FTIR spectrum of GE-PHB-PLGA formulation in comparison with base components' spectra.

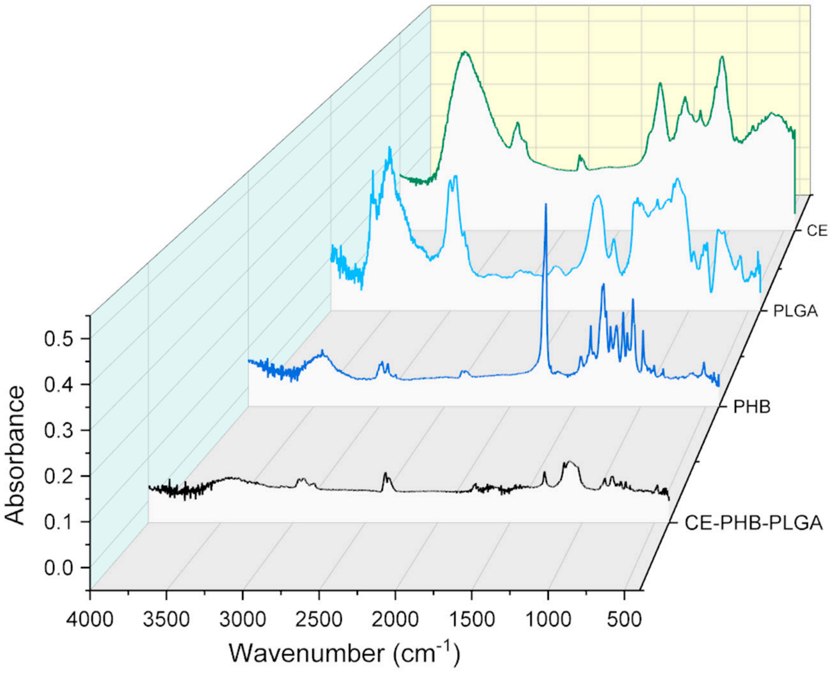

Figure S25. ATR-FTIR spectrum of CE-PHB-PLGA formulation in comparison with base components' spectra.

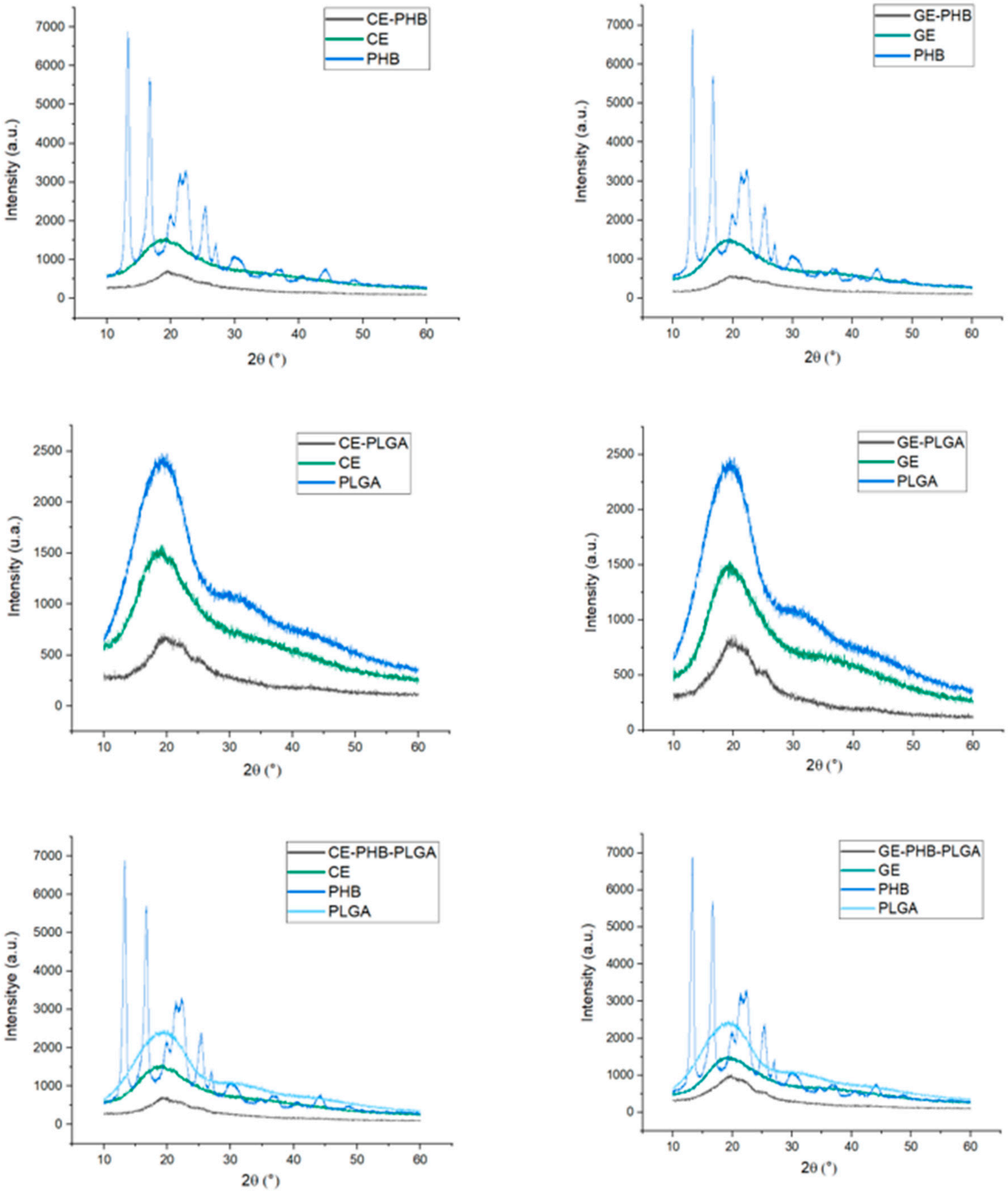

Figure S26. Diffraction patterns of formulations in comparison with base components.

3D	SE-PHB	CE-PHB	GE-PHB
8 µm x 8 µm	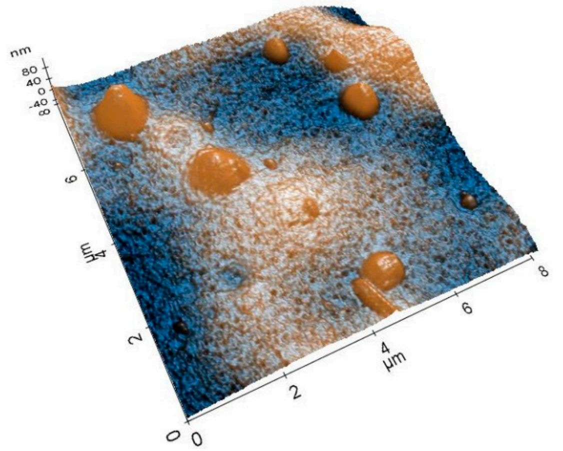	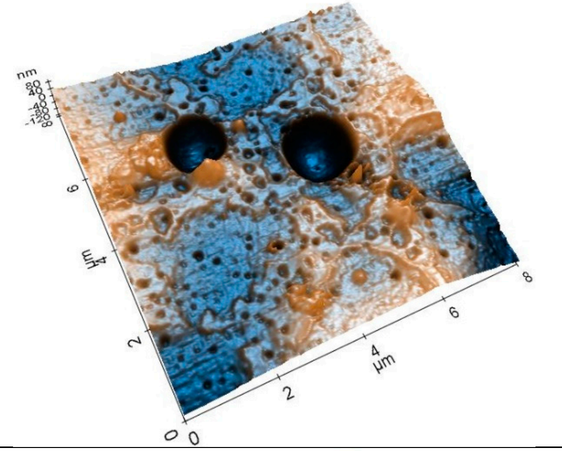	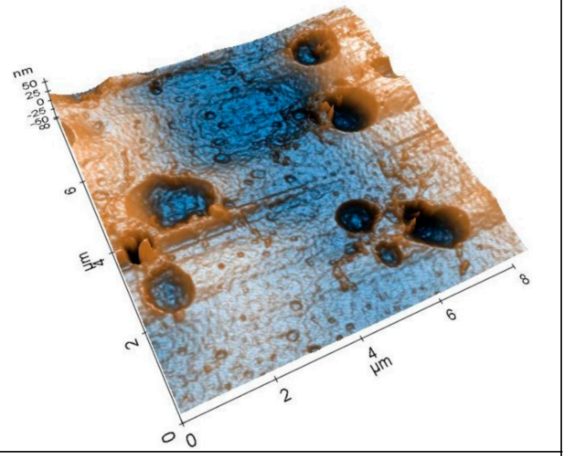
2 µm x 2 µm	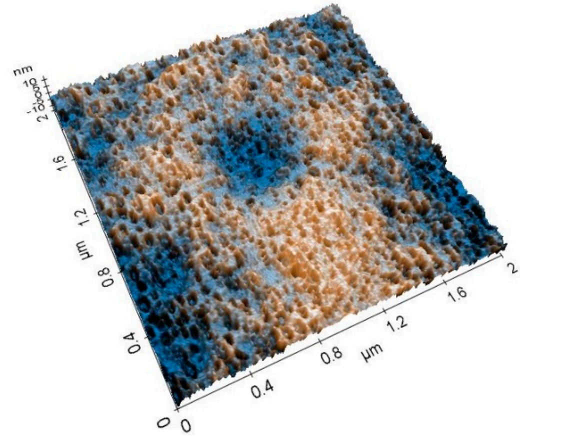	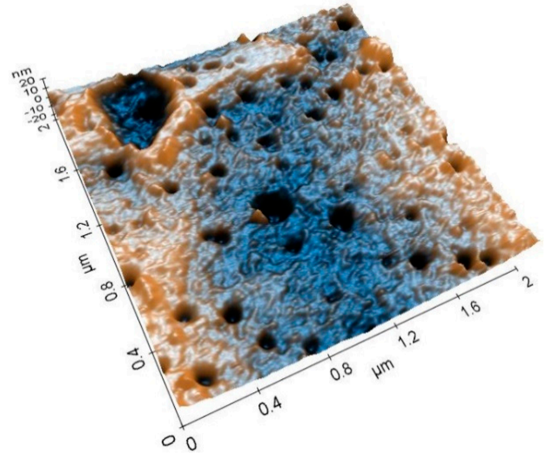	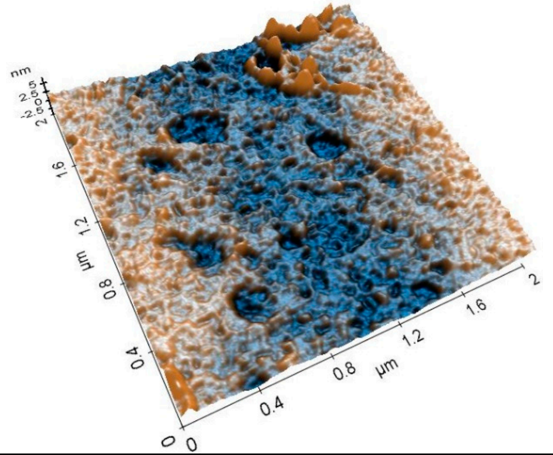

Figure S27. 3D AFM images of PHB formulations samples.

3D	SE-PLGA	CE-PLGA	GE-PLGA
8 µm x 8 µm	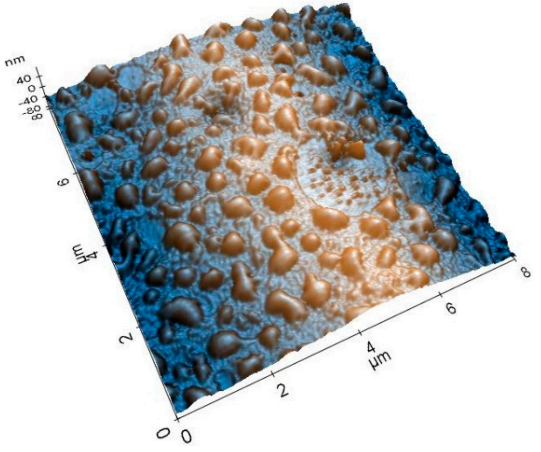	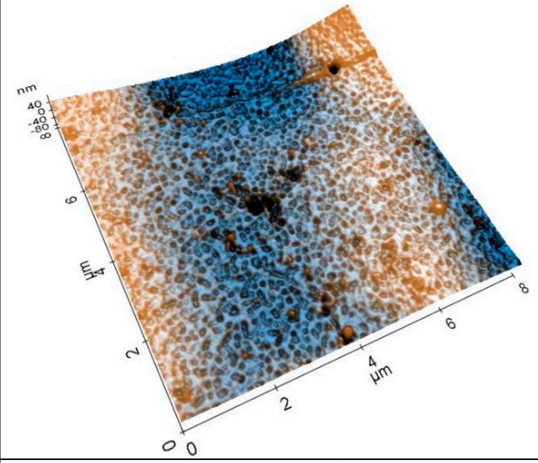	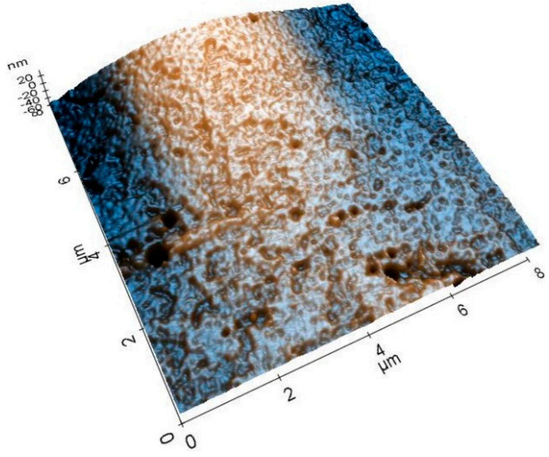
2 µm x 2 µm	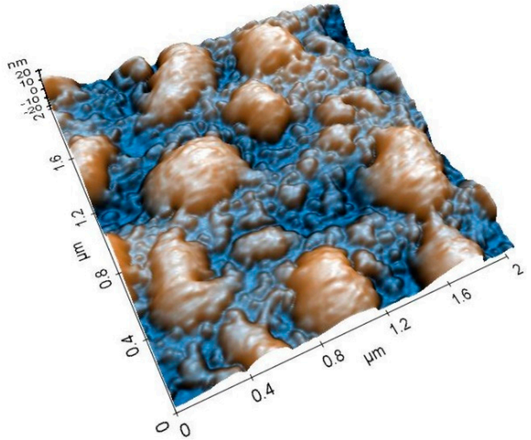	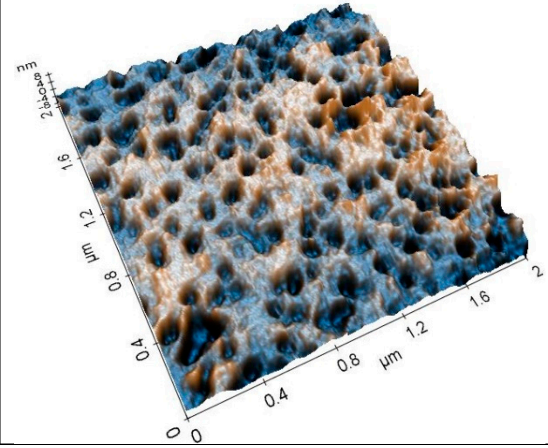	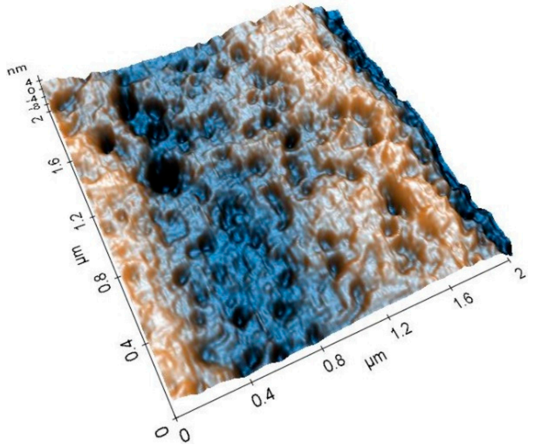

Figure S28. 3D AFM images of PLGA formulations samples.

3D	SE-PHB-PLGA	CE-PHB-PLGA	GE-PHB-PLGA
8 µm x 8 µm	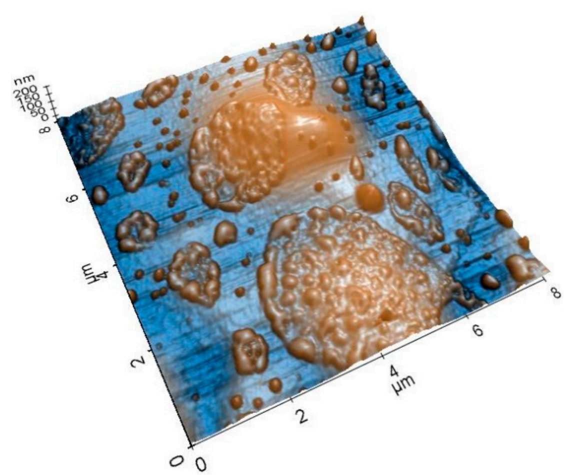	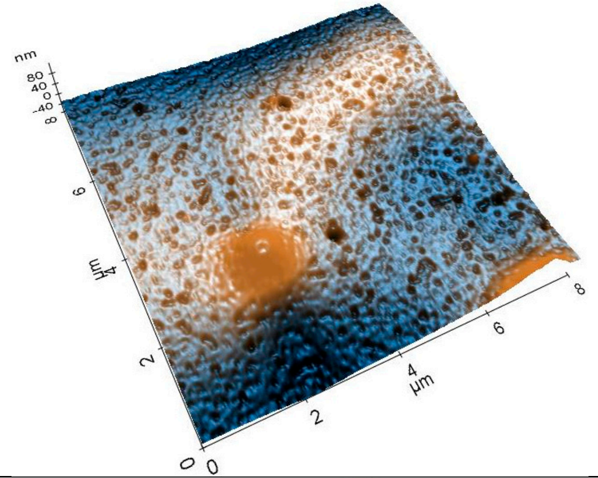	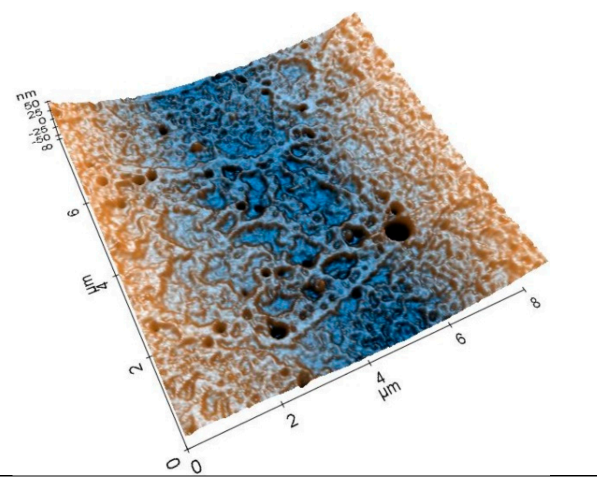
2 µm x 2 µm	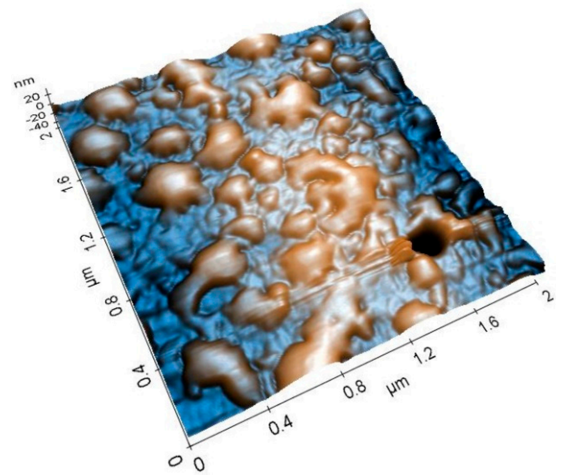	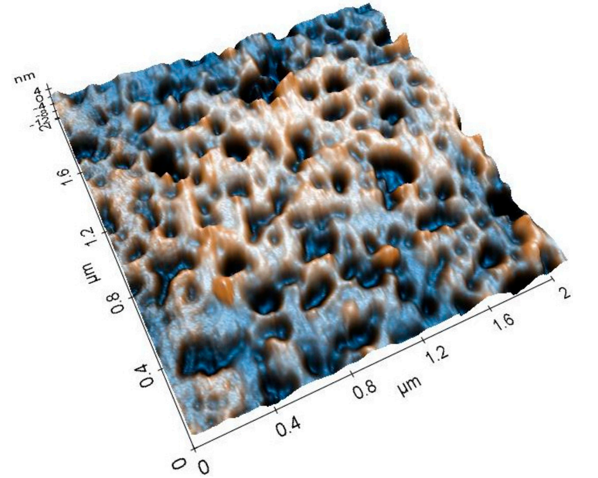	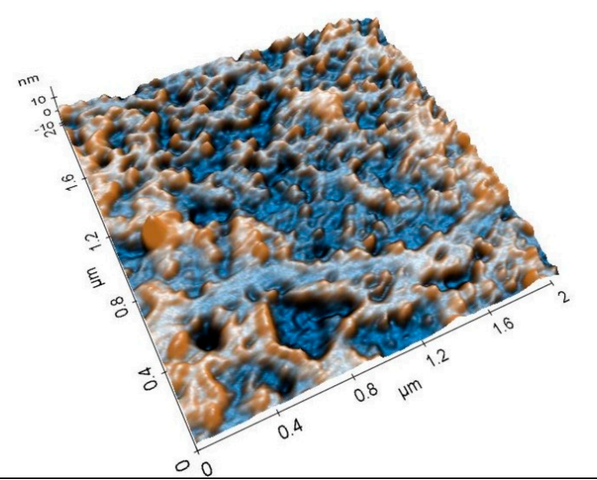

Figure S29. 3D AFM images of PHB-PLGA formulations samples.
